# Antimicrobial and Antibiofilm Activity of Acacia and Polyfloral Honey: Physicochemical Characterization and Correlation with Antibacterial Efficacy

**DOI:** 10.3390/foods15122076

**Published:** 2026-06-08

**Authors:** Alexandru Nan, Marioara Nicoleta Caraba, Mihai Mituletu, Gabi Dumitrescu, Ion Valeriu Caraba, Isabella Ionela Stoian, Adrian Sinitean, Roxana Popescu, Daniela Puscasiu

**Affiliations:** 1 Doctoral School “Engineering of Vegetable and Animal Resources”, University of Life Sciences “King Mihai I” from Timişoara, Calea Aradului 119, 300645 Timişoara, Romania; alexandru.nan@usvt.ro; 2 Faculty of Bioengineering of Animal Resources, University of Life Sciences “King Mihai I” from Timisoara, Calea Aradului 119, 300645 Timisoara, Romania; gabidumitrescu@usvt.ro (G.D.); valeriucaraba@usvt.ro (I.V.C.); 3 ANAPATMOL Research Center, “Victor Babes” University of Medicine and Pharmacy of Timisoara, E. Murgu 2, 300041 Timisoara, Romania; mihai.mituletu@umft.ro (M.M.); isabella.stoian@umft.ro (I.I.S.); popescu.roxana@umft.ro (R.P.); puscasiu.daniela@umft.ro (D.P.); 4 Faculty of Medicine, “Victor Babes” University of Medicine and Pharmacy Timisoara, E. Murgu 2, 300041 Timisoara, Romania; 5 Doctoral School, Faculty of Medicine, “Victor Babeș” University of Medicine and Pharmacy, Eftimie Murgu 2, 300041 Timisoara, Romania; 6 Department Biology, Faculty of Chemistry-Biology-Geography, West University of Timisoara, Pestalozzi 16, 300315 Timisoara, Romania; adrian.sinitean@e-uvt.ro

**Keywords:** natural product, acacia honey, polyflora honey, antibacterian, antibiofilm

## Abstract

Honey has been appreciated for its medicinal properties since ancient times; it is known as a powerful antimicrobial agent, and as a result of the increase in antibiotic resistance of various bacterial strains, honey began to be used in complementary therapies to combat microbial infections. The study aimed to identify the antimicrobial potential of two honey varieties (Acacia honey and polyflora honey) with different botanical and geographical origins on standardized bacterial strains or isolated from patients, some of which showed antibiotic resistance. The physicochemical parameters of the honey varieties analyzed were: water content, impurities, pH of honey, acidity, mineral content, reducing sugar content, total phenol content, and antioxidant capacity (DPPH). The antibacterial potential of the honey varieties was assessed based on tests to determine cell viability and the capacity to inhibit biofilm formation. The Gram-positive strains studied were *Staphylococcus aureus* (ATCC25923), *Staphylococcus aureus* MRSA (ATCC43300), *Streptococcus pneumoniae* (ATCC49619), and the Gram-negative strain was *Escherichia coli* (ATCC25922). In addition, bacterial strains isolated from the patients were *Staphylococcus aureus*, *Staphylococcus aureus* MRSA, *Streptococcus pneumoniae*, and *Escherichia coli*. The results of the microbiological tests were correlated with the physicochemical parameters, suggesting that the content of polyphenolic compounds with antioxidant activity and acidic pH may contribute to the antimicrobial potential of honey. Also, statistical analyses indicated significant differences regarding the antimicrobial potential of honey on Gram-positive versus Gram-negative bacteria, standardized versus isolated bacteria from patients, but also for antibiotic-resistant bacteria compared to the other strains studied.

## 1. Introduction

Honey is a natural food produced by bees as a result of the enzymatic transformation of floral nectar or extrafloral juices [1]. Honey is a mixture of organic and inorganic compounds, with a high carbohydrate content (80–85%), mainly simple sugars—fructose and glucose (with nutritional and medical benefits), vitamins (thiamin, niacin, riboflavin, vitamin B6, pantothenic acid), minerals (calcium, magnesium, sodium, potassium, zinc, iron, phosphorus, copper, manganese), amino acids and enzymes (amylase, invertase), antioxidant compounds, hydrogen peroxide, lysozyme, polyphenols, phenolic acids, flavonoids, methylglyoxal and peptides [2,3,4,5,6].

The organoleptic properties of honey—taste, color, and its chemical composition—are directly influenced by the bee species, its botanical origin, climatic and meteorological conditions, geographical region, and also by its processing, packaging and storage processes [7,8].

Due to its composition, honey has been used for therapeutic purposes since ancient times and continues to be used today. The therapeutic potential of honey is closely related to its chemical composition. It is known that the content of polyphenols and flavonoids is responsible for the antioxidant and antimicrobial properties of honey [9,10,11,12].

In Romania, due to the great diversity of flora, numerous types of honey are obtained, with a distinct aroma and taste, with superior qualities, rich in nutrients and substances beneficial for health. Thus, the analysis of beekeeping products, implicitly of honey with different botanical and geographical origins, was carried out by several groups of researchers, highlighting, based on the analysis of a large number of physicochemical parameters, a complete and complex composition of honey. This composition is responsible for and closely related to the antioxidant, antimicrobial, and antitumor properties, thus highlighting the therapeutic potential of honey varieties from apiaries located on the territory of Romania [13,14,15,16,17,18,19,20,21].

Bee products, including honey, are characterized by numerous therapeutic effects such as antimicrobial properties [22,23,24,25,26,27], antifungal [28], antiviral [27,29,30], anti-inflamatory [31,32,33], anticancer [34,35,36], antitrombotic [23], antiulcerose [25], antioxidant [27,37,38], hepatoprotective [39,40], immunostimulant–immunomodulatory [41,42,43], analgesic [42,44] and wound healing capacity [45]. Recently, apitherapy has focused on using bee products as a powerful adjuvant to support the management and healing of numerous types of conditions within alternative medicine practices [46].

The main bioactive compounds in honey, responsible for antioxidant and antitumor actions or for treating various types of diseases, are considered flavonoids and phenolic acids [47]. Bioactive compounds act through a variety of mechanisms to demonstrate antitumor activity, including apoptosis, tumor necrosis factor inhibition, and antiproliferative, immunomodulatory, and anti-inflammatory effects [47].

Another benefit of honey for human health is its antioxidant capacity, due to its effect on combating oxidative damage [42,48,49]. The antioxidant capacity is believed to be due to compounds in honey such as lysozyme enzymes, carotenoids and phenolic compounds, including flavonoids, which act as free radical scavengers [42].

Honey antioxidants, such as polyphenols, tannins and flavonoids, have been shown to have inhibitory activity on enzymes; by inactivating certain enzymes, pathogens can be eliminated, or possible metabolic disorders can be corrected. In the case of inhibition of enzymes such as angiotensin, tyrosinase, xanthine oxidase, α-amylase, α-glucosidase, acetylcholinesterase and lipase, a positive impact on the response to various types of diseases, such as neurodegenerative disorders, hypertension, type 2 diabetes, is identified [42,50,51]. Also, among the therapeutic applications of honey, we mention its adjuvant role in facilitating the treatment of diseases associated with oxidative stress, such as diabetes, hypertension, atherosclerosis, cancer and Alzheimer’s disease [52].

The antimicrobial properties of honey are well known, with numerous studies in this regard, factors such as nectar source, osmolarity, H_2_O_2_ content, low pH value, polyphenol and flavonoid content, 1,2-dicarbonyl compounds and bee defensin-1, processing and storage conditions being correlated with these antimicrobial properties [2,32,53,54,55,56]. Thus, the high osmolarity of honey due to its high sugar content is known to be one of the reasons why honey inhibits the development and growth of microorganisms. It is also known that natural honey has a more obvious inhibitory effect on Gram-positive cocci with medical implications, but also on the methicillin-resistant *Staphylococcus aureus* (MRSA) strain or other enterococci, compared to honey solutions [57]. A wide range of compounds with bacteriostatic and bacteriolytic effects can be identified in different types of honey. In addition, honey, due to its ability to stimulate the release of cytokines, together with its immunomodulatory and anti-inflammatory properties, contributes to the manifestation of antimicrobial potential [54].

Due to the emergence of antibiotic-resistant microbial strains and their high prevalence in various types of diseases, great pressure has been placed on the health system. As a result, the main challenge is the discovery of new therapeutic strategies, and complementary medicine that uses natural products or their phytochemical derivatives represents an excellent alternative to overcome health problems. Thus, beekeeping products, implicitly honey, could be an option in this regard, since honey, in addition to its high nutritional value, also has medicinal properties [58]. The administration of alternative treatments (natural products, e.g., honey) is reported to exhibit strong antibacterial properties against a wide range of pathogens, including methicillin-resistant *Staphylococcus aureus* (MRSA) and *Pseudomonas aeruginosa* [32,59,60].

Even though the antimicrobial potential of honey against Gram-positive and Gram-negative organisms has been extensively analyzed in vitro, the results of the studies are contradictory. Thus, some studies highlight an antimicrobial activity of different types and varieties of honey that is more evident in Gram-positive bacteria, even in some that show antibiotic resistance, compared to Gram-negative bacteria. The different antimicrobial capacity in Gram-positive bacteria compared to Gram-negative bacteria is attributed to the different cellular structure at the level of the cell wall. Gram-negative bacteria have an external membrane outside the peptidoglycan layer that makes it difficult for compounds with bacteriolytic properties to act on the cell membranes and destroy their continuity. Other studies, however, consider that Gram-positive bacteria are more resistant to honey compounds compared to Gram-negative bacteria [61,62,63,64,65].

Although the antimicrobial properties of honey have been and are being intensively researched, at this time the mechanisms of action of honey seem to be incompletely understood; therefore, further studies are necessary in this regard. Establishing correlations between the phytochemical composition of various honey varieties and antimicrobial potential is necessary to highlight the most efficient compound or compounds that act synergistically and to identify the cellular components at which they act to manifest antimicrobial capacity.

In the present study, two honey varieties with distinct characteristics were selected: acacia honey, as a representative of monofloral honey, and polyfloral honey. Acacia honey was included to enable the characterization of a variety with a single, well-defined botanical source, providing a clear reference framework for the interpretation of results. Polyfloral honey, owing to its compositional complexity and variability, reflects the diversity of floral sources and allows for the investigation of interactions among components derived from multiple botanical origins. Together, the two varieties provide a representative overview of honey as a natural product.

The aim of this study was to carry out a comprehensive characterization of two honey varieties (acacia honey and polyfloral honey) obtained from an organic apiary located in Romania, based on their palynological profile, physicochemical parameters, antimicrobial potential, and capacity to inhibit biofilm formation against standardized Gram-positive and Gram-negative bacterial strains, clinical isolates, and antibiotic-resistant strains. In addition, the study aimed to explore possible relationships between the overall physicochemical profile of honey and its antimicrobial and antibiofilm activity through statistical analyses, rather than to establish direct causal mechanisms for individual honey constituents. The original elements of this study are as follows: (i) Make a comparative analysis of two honey varieties from an organic apiary located in the western part of Romania; (ii) establish correlations between the physicochemical profile of honey and the antibacterial capacity on standardized Gram-positive and Gram-negative bacterial strains isolated from patients with antibiotic resistance, respectively; (iii) establish correlations between the physicochemical profile of honey and the antibiofilm capacity on standardized Gram-positive and Gram-negative bacterial strains, isolated from patients, respectively with antibiotic resistance; and (iv) make a comparative analysis of the antibacterial potential on standardized Gram-positive and Gram-negative bacteria versus bacteria isolated from patients versus bacteria with antibiotic resistance.

## 2. Materials and Methods

### 2.1. Honey Samples

**Figure 1:**
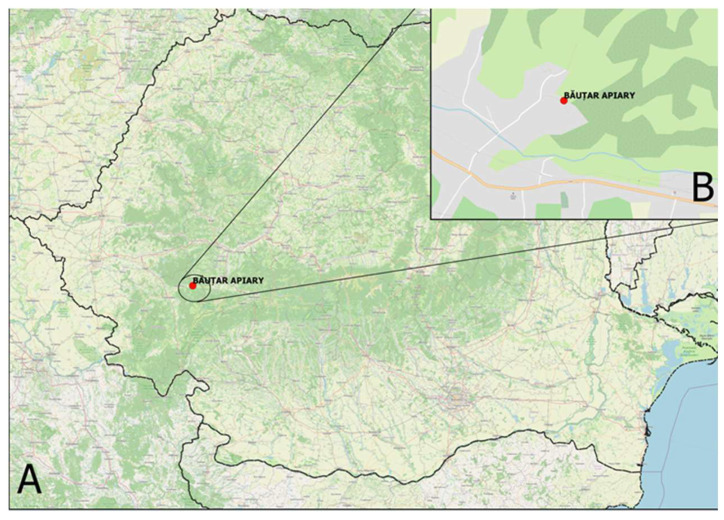
Location of the apiary (processing QGIS 3, basemap: open street map, 45°31′13.03″ N, 22°32′36.57″ E).

The types of honey analyzed were represented by acacia honey (*Acacia* sp.) and polyflora honey from an apiary located in the hill area near the town of Bautar in Caras-Severin County (45°31′13.03″ N, 22°32′36.57″ E, western part of Romania) (Figure 1). The honey samples tested for each assortment (sample A—beekeeping production of 2021 and sample B—beekeeping production of 2022) represent the single sample obtained after collecting and randomly mixing honey from 10 different samples taken from the entire quantity of acacia honey, respectively polyflora honey, obtained per season. The honey samples come from an organic apiary, with certification for honey quality, with over 7 years of experience in the beekeeping field. Acacia and polyflora honey came from the beekeeping production of 2021 and 2022. The honey samples were transported and stored in glass jars at a temperature of ≈20 °C, in a dry and dark place until the analyses were carried out. The results presented are those obtained from the analyses carried out between September–December 2021 and September–December 2022. The physicochemical analyses were carried out at the Interdisciplinary Research Platform (PCI) belonging to the “King Mihai I” University of Life Sciences in Timisoara, Romania, and the microbiological analyses were carried out at the Advanced Environmental Research Laboratories (AERL-UVT).

### 2.2. Palynological Assessment of the Honey Samples

Honey type was established by palynological examination. A 10 g sample was diluted in 20 mL distilled water, homogenized (Biosan MR-1 shaker, Riga, Latvia), and incubated at 40 °C to dissolve sugars. The mixture was centrifuged at 3500 rpm for 15 min (MPW-351RH, Warsaw, Poland), and the sediment was mounted in glycerin on glass slides and sealed with coverslips. Microscopic identification was performed using a Nikon Eclipse TE2000-U inverted microscope (Nikon Corporation, Amstelveen, The Netherlands); a total of 500 pollen grains per sample were counted and classified by size, shape, and exine structure [66,67].

### 2.3. Determination of Humidity

Moisture content was quantified by drying a 5 g subsample at 103 °C for 24 h using a conventional drying oven (Binder, Tuttlingen, Germany). Following dehydration, the samples were reweighed, and the moisture percentage was calculated using the corresponding formula:

Moisture = [(G1 − G2)/G1 − G3)] × 100 (%)

Dry matter = 100 − Moisture (%)

where G1—weight of Petri dish and sample before drying (g); G2—weight of Petri dish and sample after drying (g); and G3—weight of Petri dish (g) [68].

### 2.4. Determination of Impurities

Ten grams of honey were dissolved in 50 mL water, homogenized for 30 min with a mechanical stirrer, then filtered through qualitative filter paper. The filter paper was dried at 103 °C for 10 min, and the residue was weighed. Impurity content was calculated as follows:

I = (m1/m2) × 100 (%)

where I = impurity content (%); m1 = initial sample mass (g); m2 = mass of dried residue on filter paper (g) [47]. The results are expressed in mg/100 g honey.

### 2.5. Determination of pH

The pH was measured at 23–24 °C using an inoLab pH 730 m (Xylem Analytics, Weilheim, Germany; range: −2.000 to ±19.999; precision: ±0.05). Three grams of honey were dissolved in 30 mL water and stirred for 30 min (Holt Stirrer LM4-1002, Labor Logistics Group GmbH, Meckenheim, Germany) prior to measurement [68].

### 2.6. Determination of Acidity

Sample acidity was determined by titration. Ten grams of honey were dissolved in 50 mL water, two drops of alcoholic phenolphthalein were added as an indicator, and the mixture was shaken for 30 min (Holt IDL plate shaker, Freising, Germany), then filtered through standard filter paper. The filtrate was titrated with 0.1 N NaOH until a persistent pink coloration was maintained for 30 s. Acidity was calculated as follows:

Acidity = [(V × 0.1)/10] × 100 (mL NaOH 0.1 N/100 g honey)

where V is the volume (mL) of 0.1 N NaOH consumed [68].

### 2.7. Determination of Mineral Substance Content (Ash)

Ash content was determined gravimetrically. Three-gram samples in crucibles were gradually heated to 550 ± 25 °C in a calcination furnace (Nabertherm, Lilienthal, Germany) until a consistent white or light-gray ash was obtained. Ash content was calculated as follows:

Ash = (m − m1)/(m2 − m1) (%)

where m = mass of crucible + ash after calcination; m1 = mass of empty crucible; and m2 = mass of crucible + honey (g).

For elemental quantification, 10 mL HCl was added to the ash, transferred to glass tubes, filtered, and diluted to 50 mL with distilled water. Macro- and micro-element concentrations (mg/kg) were determined by Atomic Absorption Spectroscopy (AAS), calibrated with a multi-element standard solution (Centipur Merck, Merck KGaA, Darmstadt, Germany) [68,69].

### 2.8. Determination of Reducing Sugar

Reducing sugar levels were evaluated following the method described by Lazar et al. [70].

Three grams of honey were diluted to 200 mL with water and homogenized. A 20 mL aliquot was brought to 100 mL to obtain the working solution. Combined volumes of 20 mL copper sulfate solution, 20 mL alkaline Seignette salt solution, and 20 mL water were heated to near boiling; a total of 20 mL of working solution was added and boiled for 5 min. After cooling in a water bath, 25 mL NaCl solution was added until the solution appeared clear with a bluish-green tint, followed by 2 g sodium bicarbonate, which produced a pronounced blue precipitate. Iodine solution was added gradually until the mixture turned green; a total of 0.5 mL starch solution was then added, producing a dark-blue color. The mixture was titrated with sodium thiosulfate to a light-blue endpoint. Reducing sugar (as inverted sugar) was calculated as follows:

Inverted sugar = [(m × 10 × 5)/(m1 × 1000)] × 100 (%)

where m = inverted sugar (mg); m1 = honey mass (g); 10 = dilution factor (200 mL/20 mL); and 5 = dilution factor (100 mL/20 mL) [68]. Results are expressed as %.

### 2.9. Determination of Total Phenolic Content (PC)

One gram of honey was extracted with 10 mL of 70% ethanol for 30 min (Holt Plate Stirrer, IDL, Freising, Germany) and filtered. For colorimetric analysis, 0.5 mL of filtrate was mixed with 1.25 mL of 1:10 diluted Folin-Ciocalteu reagent (Sigma-Aldrich, Munich, Germany), incubated for 5 min at room temperature, then combined with 1 mL Na_2_CO_3_ (60 g/L) and incubated at 50 °C for 30 min (Memmert GmbH, Schwabach, Germany). Absorbance was read at 750 nm (UV-VIS Analytical Jena Specord 205, Jena, Germany), using ethanol as blank. A gallic acid calibration curve (2.5–250 µg/mL) was used; results are expressed as mg GAE/g dry matter [70].

### 2.10. Determination of Antioxidant Capacity (DPPH)

Antioxidant capacity was assessed by DPPH assay. One gram of honey was dissolved in 10 mL of 60% ethanol, homogenized, filtered, and incubated for 30 min. Absorbance was measured at 518 nm (UV-VIS Analytical Jena Specord 205, Analytik Jena AG, Jena, Germany), using 60% ethanol as a negative control and ascorbic acid as a positive control. The blank consisted of 1 mL ethanol and 2.5 mL extract, incubated for 30 min at the same wavelength. Antioxidant activity was calculated as follows:

AA (%) = 100 − [(A sample − A blank) × 100/A control]

where AA = antioxidant activity; A sample = sample absorbance; A control = DPPH absorbance; and A blank = alcohol sample absorbance [20].

The determination of all physicochemical parameters of the two analyzed honey varieties was performed in triplicate.

### 2.11. Antimicrobial Activity

Antimicrobial activity was evaluated using three concentrations of each honey variety (c1–c3), standardized reference strains (ATCC) and clinical bacterial isolates. The raw, undiluted honey collected from the apiary served as concentration c1 (100%). Honey solutions were prepared at two distinct concentrations: 80% (*w*/*w*) and 60% (*w*/*w*). The 80% solution was obtained by dissolving 80 g of honey in 20 g of water, while the 60% solution was prepared by dissolving 60 g of honey in 40 g of water. ATCC strains were sourced from the Microbiology Laboratory, AERL, West University of Timișoara; clinical isolates were obtained from the Microbiology Department, “Pius Brânzeu” Emergency Clinical Hospital, Timișoara.

#### 2.11.1. Bacterial Strains

Honey samples were tested on the following reference strains: Gram-positive strains: *Staphylococcus aureus* (ATCC 25923), *Staphylococcus aureus* MRSA (ATCC 43300), *Streptococcus pneumoniae* (ATCC 49619), and Gram-negative strains: *Escherichia coli* (ATCC 25922). The bacterial strains isolated from the patients were as follows: *Staphylococcus aureus*, *Staphylococcus aureus* MRSA, *Streptococcus pneumoniae*, and *Escherichia coli*.

#### 2.11.2. Bacterial Cultures

The standardized bacterial strains were cultured for 24 h at 37 °C in Trypticase Soy Broth (TSB) prior to testing. Clinical isolates were similarly transferred to fresh TSB medium and incubated for 24 h at 37 °C. Following incubation, the optical density (OD) of each bacterial suspension was measured at 620 nm using a BioTek Synergy/H1 microplate reader (Agilent, Santa Clara, CA, USA). Each culture was then diluted in TSB to obtain a turbidity corresponding to 0.5 McFarland standard (equivalent to 1.5 × 10^8^ CFU/mL), and the inoculum density was verified using a McFarland densitometer (Grand-Bio, Cambridge, UK). The resulting standardized inoculum was subsequently used for evaluating the antibacterial activity of the honey samples [71].

#### 2.11.3. Inhibition Rate of Biofilm Formation

Biofilm inhibition was assessed using a modified method of Knežević and Petrović, as adapted by Nan et al. [21,72].

In a 96-well microtiter plate, 100 µL of bacterial inoculum and 50 µL of honey at concentrations c1–c3 were combined and incubated at 37 °C for 24 h. Wells were washed twice with sterile 0.9% NaCl, dried at 37 °C, and stained with 200 µL of 0.4% crystal violet for 1 h at 37 °C. After rinsing, 200 µL of 30% acetic acid was added for 30 min, and absorbance was read at 570 nm (BioTek Synergy/H1). The biofilm inhibition rate was calculated as follows:

Biofilm inhibition rate (%) = (OD sample/OD control) × 100

where OD sample = treated microbial population; and OD control = untreated microbial population. All experiments were performed in triplicate.

#### 2.11.4. Bacterial Cell Viability Testing

Bacterial viability was assessed via TTC reduction, reflecting metabolic activity through dehydrogenase-mediated formazan formation. In a 96-well microplate, 100 µL of 0.5 McFarland-adjusted TSB culture and 50 µL of honey at c1–c3 were incubated at 37 °C for 24 h at 120 rpm. Then, 50 µL of 0.5% TTC was added and incubated for a further 2 h under the same conditions. Absorbance was measured at 460 nm (BioTek Synergy/H1). Inhibition rate was calculated as follows:

Rate of inhibition (%) = [(OD control − OD sample)/OD control] × 100

where OD sample = treated microbial population; and OD control = untreated microbial population [73,74]. All experiments were conducted in triplicate.

### 2.12. Statistical Analysis

Mann–Whitney U tests were applied to determine inter-year differences in antimicrobial activity for Acacia honey, with *p*-values being adjusted for multiple comparisons with the Benjamini–Hochberg false discovery rate (FDR) correction. The same approach was used for determining inter-year differences for polyfloral honey and differences in antimicrobial activity for honey from different sources (Acacia vs. polyfloral, pooled data for all years). Next, we aimed to determine how the antimicrobial effect of honey changes as its dose (or concentration) increases. Since our experiment involved only three concentration levels, classical dose-response modeling (e.g., nonlinear logistic or Hill models) for antimicrobial activity was not statistically reliable. As a result, we first used Spearman’s rho correlations to determine the monotonic association between honey concentration and antimicrobial effect. The strength of correlations was interpreted according to the absolute value of Spearman’s correlation coefficient as follows: very weak (0.00–0.19), weak (0.20–0.39), moderate (0.40–0.59), strong (0.60–0.79), and very strong (0.80–1.00). Strong associations (with a correlation coefficient above 0.6) were considered indicative of a biologically relevant trend. For these relationships, linear regression was subsequently applied to estimate the direction and magnitude of the dose-response effect. This two-step approach was adopted as an exploratory strategy to identify meaningful concentration-dependent patterns while minimizing overinterpretation of sparse data.

We compared the physicochemical parameters of Acacia honey and polyfloral honey using Mann–Whitney U tests with Benjamini–Hochberg correction (as described above) and pooled data from 2021–2022.

Next, bivariate correlation analysis (Spearman) was performed to determine the relationships between the honey physicochemical composition (e.g., acidity, pH, polyphenolic content) and its biological efficacy. To assess differences in antimicrobial and antibiofilm efficacy among Acacia and polyfloral honeys harvested in 2021 and 2022, a one-way analysis of variance (ANOVA) was applied to mean inhibition and bactericidal values. Homogeneity of variances was verified using Levene’s test. When significant differences were detected, Tukey’s Honestly Significant Difference (HSD) post hoc tests were applied, with all pairwise comparisons performed relative to the sample with the lowest observed mean value. Statistical significance was predefined at a level of *p* < 0.05.

## 3. Results

### 3.1. Palynological Analysis

The honey samples were subjected to palynological analysis to establish their botanical origin; thus, in Table 1, based on the microscopic analysis of pollen grains, the determination values for both honey assortments are shown. Sample A represents Acacia honey from the beekeeping production of 2021; at this level, a percentage of 47.23% of Acacia pollen grains was recorded, which led to its inclusion in this assortment. In the case of sample B, from the beekeeping production of 2022, based on the percentage of 42.57% Acacia pollen grains, the sample was included in this assortment. Honey samples C and D are represented by honey samples from the beekeeping productions of 2021 and 2022; based on the analysis of pollen grains, the two honey samples were included in the polyfloral honey assortment. The highest share of pollen grains for polyfloral honey samples belonged to the Astereaceae family, with values of 26.42% (polyfloral honey 2021) and 21.66% (polyfloral honey 2022).

### 3.2. Antimicrobial Activities of Acacia and Polyfloral Honey

The results regarding the inhibition of biofilm formation outcomes for the analyzed bacterial strains and all tested honey concentrations (60%, 80%, and 100%) are given in Table 2 and Table 3, respectively. The capacity of honey to inhibit biofilm formation across all years, honey types, and concentrations varied widely depending on the bacterial strain. The largest range of variation was observed for *Staphylococcus aureus* ATCC 25923, while the narrowest range was recorded for *Streptococcus pneumoniae* clinical isolates. The measured values ranged for (i) for *Staphylococcus aureus* ATCC 25923, between −25.97% for Acacia honey (2022) at 100% concentration and 27.20% for polyfloral honey (2021) at 100% concentration; (ii) for methicillin-resistant *Staphylococcus aureus* ATCC 25923, between 0.90% for Acacia honey (2021) at 60% concentration and 24.80% for polyfloral honey (2022) at 100% concentration; (iii) for *Staphylococcus aureus* clinical isolate, between 23.31% for polyfloral honey (2022) at 60% concentration and 46.78% for polyfloral honey (2021) at 100% concentration; (iv) for methicillin-resistant *Staphylococcus aureus* clinical isolate, between −10.67% for polyfloral honey (2021) at 60% concentration and 31.61% for polyfloral honey (2022) at 100% concentration; (v) for *Streptococcus pneumoniae* ATCC 49619, between −7.77% for polyfloral honey (2021) at 100% concentration and 12.88% for polyfloral honey (2022) at 100% concentration; (v) for *Streptococcus pneumoniae* clinical isolate, between −26.40% for Acacia honey (2021) at 100% concentration and 12.88% for Acacia honey (2021) at 80% concentration; (vi) for *Streptococcus pneumoniae* clinical isolate, between −24.87% for Acacia honey (2022) at 100% concentration and −8.34% for Acacia honey (2021) at 80% concentration; (vii) for *Escherichia coli* ATCC 25922, between 14.77% for Acacia honey (2022) at 60% concentration and 32.70% for polyfloral honey (2022) at 100% concentration; and (viii) for *Escherichia coli* clinical isolate between 22.23% for Acacia honey (2022) at 80% concentration and 39.95% for polyfloral honey (2021) at 100% concentration.

**Figure 2:**
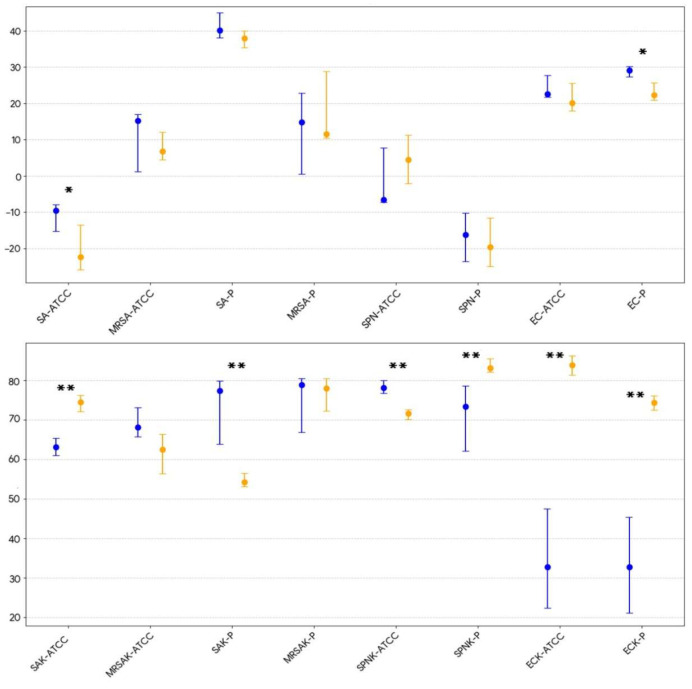
Year-to-year variation in antimicrobial activity of Acacia honey: biofilm inhibition (**upper panel**) and bactericidal activity (**lower panel**). Values are presented as median (points) with error bars representing the lower and upper percentiles. Blue values denote samples collected in 2021, whereas orange values represent samples collected in 2022. Asterisks (*) indicate significant inter-year differences (Mann–Whitney U test with Benjamini–Hochberg correction, ** *p* < 0.01, * *p* < 0.05). SA-ATCC, biofilm inhibition capacity (%) against *Staphylococcus aureus* ATCC; MRSA-ATCC, biofilm inhibition capacity (%) against methicillin-resistant *Staphylococcus aureus* ATCC; SA-P, biofilm inhibition capacity (%) against *Staphylococcus aureus* clinical isolate; MRSA-P, biofilm inhibition capacity (%) against methicillin-resistant *Staphylococcus aureus* clinical isolate; SPN-ATCC, biofilm inhibition capacity (%) against *Streptococcus pneumoniae* ATCC; SPN-P, biofilm inhibition capacity (%) against *Streptococcus pneumoniae* clinical isolate; EC-ATCC, biofilm inhibition capacity (%) against *Escherichia coli* ATCC; EC-P, biofilm inhibition capacity (%) against *Escherichia coli* clinical isolate; SAK-ATCC, bactericidal activity (%) against *Staphylococcus aureus* ATCC; MRSAK-ATCC, bactericidal activity (%) against methicillin-resistant *Staphylococcus aureus* ATCC; SAK-P, bactericidal activity (%) against *Staphylococcus aureus* clinical isolate; MRSAK-P, bactericidal activity (%) against methicillin-resistant *Staphylococcus aureus* clinical isolate; SPNK-ATCC, bactericidal activity (%) against *Streptococcus pneumoniae* ATCC; SPNK-P, bactericidal activity (%) against *Streptococcus pneumoniae* clinical isolate; ECK-ATCC, bactericidal activity (%) against *Escherichia coli* ATCC; ECK-P, bactericidal activity (%) against *Escherichia coli* clinical isolate.

Across all years, honey types, and concentrations, bactericidal activity varied widely depending on the bacterial strain. The broadest ranges were observed for *Streptococcus pneumoniae* clinical isolates and *Escherichia coli* ATCC 25922, whereas the narrowest range occurred for methicillin-resistant *Staphylococcus aureus* clinical isolates. The measured values ranged as follows: (i) for *Staphylococcus aureus* ATCC 25923, between −50.44% for polyfloral honey (2021) at 60% concentration and 78.49% for Acacia honey (2022) at 100% concentration; (ii) for methicillin-resistant *Staphylococcus aureus* ATCC 25923, between 51.92% for polyfloral honey (2022) at 60% concentration and 74.67% for Acacia honey (2021) at 100% concentration; (iii) for *Staphylococcus aureus* clinical isolate, between 51.05% for Acacia honey (2022) at 60% concentration and 79.89% for Acacia honey (2021) at 100% concentration; (iv) for methicillin-resistant *Staphylococcus aureus* clinical isolate, between 64.33% for Acacia honey (2021) at 60% concentration and 81.13% for Acacia honey (2022) at 100% concentration; (v) for *Streptococcus pneumoniae* ATCC 49619, between 31.06% for polyfloral honey (2021) at 60% concentration and 80.00% for Acacia honey (2021) at 100% concentration; (vi) for *Streptococcus pneumoniae* clinical isolate, between 15.06% for polyfloral honey (2021) at 60% concentration and 84.98% for Acacia honey (2022) at 100% concentration; (vii) for *Escherichia coli* ATCC 25922, between 19.67% for Acacia honey (2021) at 60% concentration and 88.71% for Acacia honey (2022) at 100% concentration; and (viii) for *Escherichia coli* clinical isolate (ECK-P), between 19.87% for Acacia honey (2021) at 60% concentration and 79.70% for polyfloral honey (2022) at 100% concentration (Figure 2 and Table 2).

### 3.3. Year-to-Year Variation in Antimicrobial Activity of Honey

**Figure 3:**
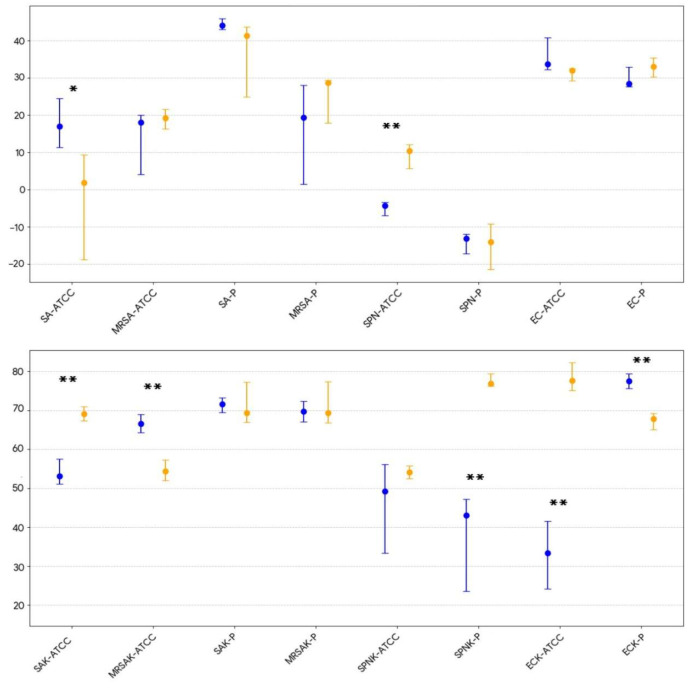
Year-to-year variation in antimicrobial activity of polyfloral honey: biofilm inhibition (**upper panel**) and bactericidal activity (**lower panel**). Values are presented as median (points) with error bars representing the lower and upper percentiles. Blue values denote samples collected in 2021, whereas orange values represent samples collected in 2022. Asterisks (*) indicate significant inter-year differences (Mann–Whitney U test with Benjamini–Hochberg correction, ** *p* < 0.01, * *p* < 0.05). SA-ATCC, biofilm inhibition capacity (%) against *Staphylococcus aureus* ATCC; MRSA-ATCC, biofilm inhibition capacity (%) against methicillin-resistant *Staphylococcus aureus* ATCC; SA-P, biofilm inhibition capacity (%) against *Staphylococcus aureus* clinical isolate; MRSA-P, biofilm inhibition capacity (%) against methicillin-resistant *Staphylococcus aureus* clinical isolate; SPN-ATCC, biofilm inhibition capacity (%) against *Streptococcus pneumoniae* ATCC; SPN-P, biofilm inhibition capacity (%) against *Streptococcus pneumoniae* clinical isolate; EC-ATCC, biofilm inhibition capacity (%) against *Escherichia coli* ATCC; EC-P, biofilm inhibition capacity (%) against *Escherichia coli* clinical isolate; SAK-ATCC, bactericidal activity (%) against *Staphylococcus aureus* ATCC; MRSAK-ATCC, bactericidal activity (%) against methicillin-resistant *Staphylococcus aureus* ATCC; SAK-P, bactericidal activity (%) against *Staphylococcus aureus* clinical isolate; MRSAK-P, bactericidal activity (%) against methicillin-resistant *Staphylococcus aureus* clinical isolate; SPNK-ATCC, bactericidal activity (%) against *Streptococcus pneumoniae* ATCC; SPNK-P, bactericidal activity (%) against *Streptococcus pneumoniae* clinical isolate; ECK-ATCC, bactericidal activity (%) against *Escherichia coli* ATCC; ECK-P, bactericidal activity (%) against *Escherichia coli* clinical isolate.

Annual variation in the antimicrobial activity of *Acacia* honey and polyfloral honey —only the results obtained for 100% honey concentration—are presented in Figure 2 and Figure 3, respectively, with biofilm inhibition capacity displayed in the upper panels and bactericidal activity in the lower panels. *Acacia* honey samples from 2021 showed a significantly higher capacity to inhibit *Staphylococcus aureus* ATCC 25923 biofilm formation than those collected in 2022. A similar situation was encountered for the *Escherichia coli* clinical isolate, whereas no significant inter-year differences were observed for the other bacteria.

Bactericidal activity of Acacia honey, by contrast, showed more pronounced differences, but without showing a clear pattern in *Staphylococcus aureus* and *Streptococcus pneumoniae* strains. Thus, samples collected in 2021 exhibited a significantly higher bactericidal activity versus those produced in 2022 against *Staphylococcus aureus* ATCC 25923 and clinical isolates of *Streptococcus pneumoniae*. The opposite pattern was observed for the *Staphylococcus aureus* clinical isolate and *Streptococcus pneumoniae* ATCC 49619. However, *E. coli* showed a homogeneous trend, with a significantly increased bactericidal activity seen in 2021 irrespective of bacterial strain.

We observed that polyfloral honey samples collected in 2022 exhibited a significantly greater capacity to inhibit biofilm formation by *Staphylococcus aureus* ATCC 25923 than those harvested the following year. In contrast, a significantly lower inhibitory effect on *Streptococcus pneumoniae* ATCC 49619 was detected in samples obtained in 2021. Polyfloral honey harvested in 2022 exerted a significantly higher bactericidal activity against *Staphylococcus aureus* ATCC 25923 and *Streptococcus pneumoniae* ATCC 49619. An inverse pattern was observed for methicillin-resistant *Staphylococcus aureus* ATCC 25923. We also note that, unlike Acacia honey, polyfloral honey induced distinct effects against *Escherichia coli*: samples collected in 2021 had a significantly stronger effect against strain ATCC, but a significantly lower impact on clinical isolates.

### 3.4. Antimicrobial Activity of Honey with Different Botanical Origins

**Figure 4:**
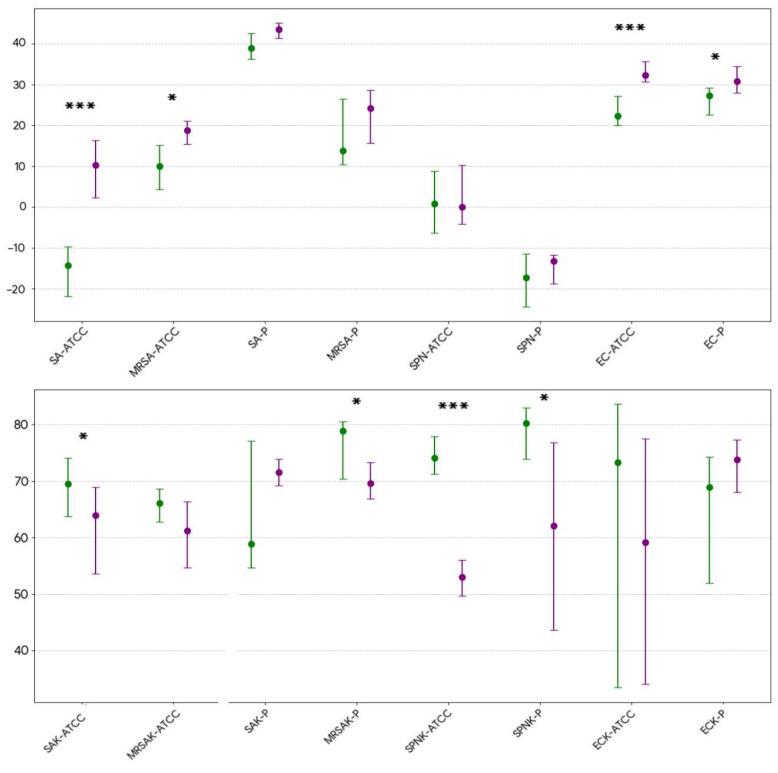
Comparative antimicrobial activity of Acacia honey and polyfloral honey: biofilm inhibition (**upper panel**) and bactericidal activity (**lower panel**). Values are presented as median (points) with error bars representing the lower and upper percentiles. Green values denote samples collected in 2021, whereas purple values represent samples collected in 2022. Asterisks (*) indicate significant differences between honey samples obtained from different plant sources (Mann–Whitney U test with Benjamini–Hochberg correction, *** *p* < 0.001, * *p* < 0.05). SA-ATCC, biofilm inhibition capacity (%) against *Staphylococcus aureus* ATCC; MRSA-ATCC, biofilm inhibition capacity (%) against methicillin-resistant *Staphylococcus aureus* ATCC; SA-P, biofilm inhibition capacity (%) against *Staphylococcus aureus* clinical isolate; MRSA-P, biofilm inhibition capacity (%) against methicillin-resistant *Staphylococcus aureus* clinical isolate; SPN-ATCC, biofilm inhibition capacity (%) against *Streptococcus pneumoniae* ATCC; SPN-P, biofilm inhibition capacity (%) against *Streptococcus pneumoniae* clinical isolate; EC-ATCC, biofilm inhibition capacity (%) against *Escherichia coli* ATCC; EC-P, biofilm inhibition capacity (%) against *Escherichia coli* clinical isolate; SAK-ATCC, bactericidal activity (%) against *Staphylococcus aureus* ATCC; MRSAK-ATCC, bactericidal activity (%) against methicillin-resistant *Staphylococcus aureus* ATCC; SAK-P, bactericidal activity (%) against *Staphylococcus aureus* clinical isolate; MRSAK-P, bactericidal activity (%) against methicillin-resistant *Staphylococcus aureus* clinical isolate; SPNK-ATCC, bactericidal activity (%) against *Streptococcus pneumoniae* ATCC; SPNK-P, bactericidal activity (%) against *Streptococcus pneumoniae* clinical isolate; ECK-ATCC, bactericidal activity (%)against *Escherichia coli* ATCC; ECK-P, bactericidal activity (%) against *Escherichia coli* clinical isolate.

The measured values for antimicrobial properties of honeys derived from different plant species—only the results obtained for 100% honey concentration—are given in Figure 4. It was found that polyfloral honey exerts a stronger inhibitory effect on biofilm formation for the bacterial strains analyzed. Significant differences were found for *Staphylococcus aureus* ATCC 25923, methicillin-resistant *Staphylococcus aureus* ATCC 43300, and both strains of *E. coli* ATCC 25922. However, Acacia honey tended to exert a stronger bactericidal action. Significant effects were detected again for *Staphylococcus aureus* ATCC 25923 and *Staphylococcus aureus* ATCC 43300, as well as for both strains of *Streptococcus pneumoniae* ATCC 49619.

### 3.5. Dose-Response Curves

Table 4 summarizes the Spearman’s rank correlation between honey concentration and its antibacterial activity, using pooled data from the 2021–2022 period. Acacia honey demonstrated strong to very strong associations between dose and antimicrobial activity against all methicillin-resistant bacterial strains. Moderate positive correlations were also identified for biofilm inhibition capacity against *E. coli*. In contrast, there was a strong inverse relationship between Acacia honey concentration and its ability to inhibit biofilm formation in the case of a standard (ATCC) strain of *Staphylococcus aureus*.

A trend towards strong positive associations between dose and overall antimicrobial activity (both biofilm inhibition capacity and bactericidal activity) against methicillin-resistant pathogens was observed in the case of polyfloral honey; the only exception was bactericidal activity against methicillin-resistant *Staphylococcus aureus* ATCC. There were also direct (moderate to strong) correlations between honey concentration and its ability to inhibit biofilm formation in the case of standard (ATCC) and clinical strains of *Staphylococcus aureus*, and a clinical isolate of *E. coli*. Similar relationships were observed for bactericidal activity against a clinical isolate of *Staphylococcus aureus* and a clinical isolate of *Streptococcus pneumoniae*. The other associations were not statistically significant, irrespective of honey origin.

Table 5 displays the regression equations and the corresponding R^2^ values that characterize the antibacterial activity of Acacia and polyfloral honey. Globally speaking, Acacia honey exhibited a more pronounced dose-dependent antimicrobial effect. Biofilm inhibition capacity was more sensitive to changes in honey concentration, especially in the case of Acacia honey applied to methicillin-resistant strains. This suggests that relatively small variations in Acacia honey concentration produced marked changes in antibiofilm efficacy against resistant bacteria, reflecting a strong concentration–response relationship for this endpoint. The most predictable and linear effect was observed in methicillin-resistant clinical strains.

### 3.6. Statistical Profiling of Antimicrobial Efficacy

**Figure 5:**
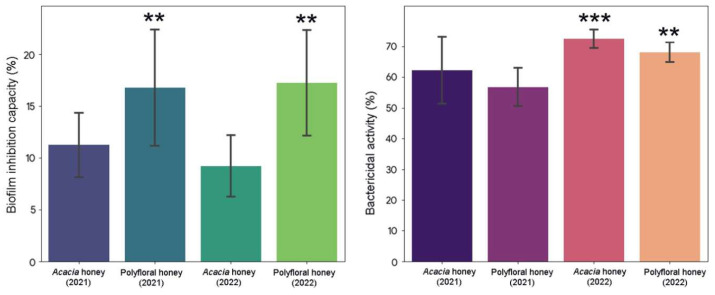
Biofilm inhibition capacity (**left panel**) and bactericidal activity (**right panel**) of Acacia and polyfloral honeys harvested in 2021 and 2022. Values are given as representing mean (bars) with one standard deviation (error bars). Asterisks (*) indicate significant differences between honey samples obtained from different sources and years (Tukey’s HSD tests, *** *p* < 0.001, ** *p* < 0.01).

Figure 5 illustrates the biofilm inhibition capacity and bactericidal activity of *Acacia* and polyfloral honeys obtained from production in 2021 and 2022—only the results obtained for 100% honey concentration. Data sets for both variables showed homogeneity of variance (*p* ≥ 0072), confirming the reliability of the parametric tests. ANOVA analysis revealed significant differences between the samples for both biofilm inhibition (*p* < 0.001) and bactericidal effect (*p* < 0.001). This proves that the specific honey type and harvest year are decisive factors in efficacy.

The lowest mean biofilm inhibition was observed for Acacia honey harvested in 2022. Using this sample as the reference, polyfloral honey from both 2021 and 2022 demonstrated a significantly greater ability to inhibit bacterial biofilm formation. In terms of bactericidal activity, polyfloral honey harvested in 2021 exhibited the lowest efficacy, whereas significantly higher bactericidal effects were recorded for honey samples collected in 2022.

### 3.7. Physicochemical Profile of Different Honey Types

Table 6 presents the physicochemical parameters of each honey sample analyzed separately according to honey type and harvest year (Acacia 2021, polyfloral 2021, Acacia 2022, and polyfloral 2022). The broadest ranges (in terms of relative variation) were observed for iron and potassium, whereas the narrowest range occurred for total reducing sugars. The measured values ranged as follows: (i) for water content, between 16.36% for polyfloral honey (2022) and 18.41% for polyfloral honey (2021); (ii) for impurity content, between 0.40% for Acacia honey (2022) and 0.69% for polyfloral honey (2021); (iii) for ash content, between 0.44% for Acacia honey (2022) and 0.55% for Acacia honey (2021); (iv) for total acidity, between 1.40 meq/kg for Acacia honey (2022) and 9.13 meq/kg for polyfloral honey (2021); (v) for pH, between 3.00 for Acacia honey (2021) and 3.38 for polyfloral honey (2022); (vi) for reducing sugars, between 66.81% for Acacia honey (2021) and 73.05% for polyfloral honey (2021); (vii) for antioxidant activity (DPPH), between 40.26% for Acacia honey (2021) and 57.44% for polyfloral honey (2022); (viii) for polyphenol content, between 805.67 mg GAE/kg for Acacia honey (2021) and 1286.35 mg GAE/kg for polyfloral honey (2021); (ix) for cadmium, between 0.022 mg/kg for polyfloral honey (2022) and 0.069 mg/kg for polyfloral honey (2021); (x) for copper, between 0.242 mg/kg for polyfloral honey (2021) and 0.397 mg/kg for Acacia honey (2021); (xi) for nickel, between 0.231 mg/kg for Acacia honey (2021) and 0.779 mg/kg for Acacia honey (2022); (xii) for chromium, between 0.058 mg/kg for polyfloral honey (2022) and 0.120 mg/kg for Acacia honey (2021); (xiii) for lead, between 0.051 mg/kg for Acacia honey (2021) and 0.170 mg/kg for polyfloral honey (2022); (xiv) for zinc, between 3.49 mg/kg for polyfloral honey (2022) and 10.76 mg/kg for Acacia honey (2021); (xv) for iron, between 1.08 mg/kg for Acacia honey (2022) and 19.03 mg/kg for polyfloral honey (2022); (xvi) for manganese, between 0.270 mg/kg for Acacia honey (2022) and 0.786 mg/kg for polyfloral honey (2021); (xvii) for calcium, between 48.04 mg/kg for polyfloral honey (2021) and 274.30 mg/kg for polyfloral honey (2022); (xviii) for magnesium, between 18.36 mg/kg for Acacia honey (2022) and 56.67 mg/kg for polyfloral honey (2022); and (xix) for Potassium (K), between 109.82 mg/kg for Acacia honey (2021) and 946.29 mg/kg for polyfloral honey (2022).

Table 7 summarizes the pooled data obtained for the two honey types across both years (2021–2022) and provides the overall statistical comparison between Acacia and polyfloral honey independent of harvest year. Polyfloral honey exhibited significantly higher impurity content, suggesting increased environmental and botanical complexity associated with multifloral nectar sources. Moreover, this type of honey—Acacia revealed a significantly higher pH than polyfloral honey. In contrast, polyfloral honey displayed higher concentrations of reducing sugars. Moreover, it demonstrated significantly higher DPPH radical scavenging activity and total polyphenol content.

With respect to their mineral content, polyfloral honey contained significantly higher concentrations of lead and manganese, whereas chromium levels were significantly elevated in Acacia honey. In addition, magnesium concentrations were significantly elevated in polyfloral honey, reinforcing its richer mineral profile. Despite these differences, all measured values remained within accepted safety limits for honey. No significant differences were determined for the other physicochemical parameters investigated.

### 3.8. Correlations Between Antimicrobial Activity and Physicochemical Parameters

**Figure 6:**
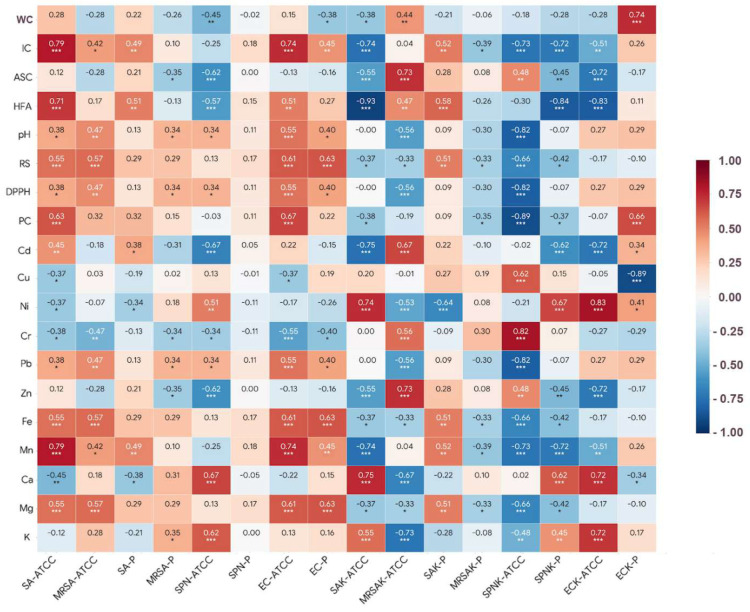
Spearman’s correlation matrix of physicochemical properties of honey and antimicrobial activity. Positive correlations are shown in blue and negative correlations in red. Asterisks (*) indicate significant correlations (Spearman’s correlation, *** *p* < 0.001, ** *p* < 0.01, * *p* < 0.05). WC, water content; IC, impurity content; ASC, ash content; HFA, total acidity of honey; RS, total reducing sugar content; DPPH, antioxidant activity as radical scavenging capacity; PC, polyphenol content; Cd, cadmium content; Cu, copper content; Ni, nickel content; Cr, chromium content; Pb, lead content; Zn, zinc content; Fe, iron content; Mn, manganese content; Ca, calcium content; Mg, magnesium content; K, potassium content; SA-ATCC, biofilm inhibition capacity (%) against *Staphylococcus aureus* ATCC; MRSA-ATCC, biofilm inhibition capacity (%) against methicillin-resistant *Staphylococcus aureus* ATCC; SA-P, biofilm inhibition capacity (%) against *Staphylococcus aureus* clinical isolate; MRSA-P, biofilm inhibition capacity (%) against methicillin-resistant *Staphylococcus aureus* clinical isolate; SPN-ATCC, biofilm inhibition capacity (%) against *Streptococcus pneumoniae* ATCC; SPN-P, biofilm inhibition capacity (%) against *Streptococcus pneumoniae* clinical isolate; EC-ATCC, biofilm inhibition capacity (%) against *Escherichia coli* ATCC; EC-P, biofilm inhibition capacity (%) against *Escherichia coli* clinical isolate; SAK-ATCC, bactericidal activity (%) against *Staphylococcus aureus* ATCC; MRSAK-ATCC, bactericidal activity (%) against methicillin-resistant *Staphylococcus aureus* ATCC; SAK-P, bactericidal activity (%) against *Staphylococcus aureus* clinical isolate; MRSAK-P, bactericidal activity (%) against methicillin-resistant *Staphylococcus aureus* clinical isolate; SPNK-ATCC, bactericidal activity (%) *against Streptococcus pneumoniae* ATCC; SPNK-P, bactericidal activity (%) against *Streptococcus pneumoniae* clinical isolate; ECK-ATCC, bactericidal activity (%) against *Escherichia coli* ATCC; ECK-P, bactericidal activity (%) against *Escherichia coli* clinical isolate.

The correlation analysis presented in this section was intended as an exploratory statistical approach to identify possible associations between the overall physicochemical profile of honey and its antimicrobial activity, without implying direct causality between individual parameters and antibacterial effects. Figure 6 presents the heat map of Spearman’s rank correlations between physicochemical parameters and antibacterial activity, including antibiofilm capacity, providing a global perspective on the honey varieties from the Bautar location. Beyond their established role as indicators of product quality and ecological integrity of the area, physicochemical parameters can be used, based on the identified correlations, to extrapolate the microbiological efficiency of honey. Thus, our results suggest that an optimal physicochemical profile not only reflects a clean environment, but also constitutes a viable predictor for antimicrobial potential, allowing for a complex and predictive characterization of the biological value of honey from this region.

The ability of honey to inhibit biofilm formation in the standard strain of *Staphylococcus aureus* showed strong positive correlations with impurity content, total acidity, polyphenol content and iron, manganese, and magnesium levels. Inverse associations were detected for calcium, copper, nickel, and chromium. Biofilm inhibition of methicillin-resistant *Staphylococcus aureus* ATCC was moderately to strongly positively correlated with impurity content, total acidity, polyphenols, manganese, magnesium, and iron, but inversely associated with chromium levels. Inhibition of biofilm formation in clinical *Staphylococcus aureus* isolates showed weak to moderate relationships; that is, direct associations with impurity content, total acidity, cadmium, and manganese, and negative associations with nickel and calcium levels. Similarly, honey-mediated inhibition of biofilm formation in the case of a methicillin-resistant clinical isolate revealed only moderate relationships. These correlations were positive with honey pH, antioxidant activity, lead, and potassium, and negative with its ash, nickel, and calcium content.

Antibiofilm activity against the standard strain (ATCC) of *Streptococcus pneumoniae* correlated directly with pH, antioxidant activity, lead, calcium, nickel, and potassium, but inversely with water, ash, cadmium, chromium, and zinc content, without a clear overall pattern in correlation strength or direction. In contrast, no significant associations existed for the clinical isolate of this bacterium. Percentage inhibition of biofilm formation by the standard strain of *Escherichia coli* was positively correlated (moderate to strong) with impurity content, total acidity, reduced sugars, antioxidant capacity, and lead, iron, manganese, and magnesium levels while showing negative correlations with chromium and copper. For the patient-derived isolate, it exhibited direct associations with impurity content, total acidity, pH, reduced sugars, antioxidant capacity, lead, iron, manganese, calcium, and potassium, and indirect associations with water, chromium, and magnesium content.

Bactericidal effect against *Staphylococcus aureus* ATCC 25923 showed positive associations with nickel, calcium, and potassium, while negative associations were found for water content, impurity content, ash content, total acidity, lead, and cadmium, with most relationships being moderate to strong. The methicillin-resistant standard strain, by contrast, correlated directly with water, ash, cadmium, chromium, and zinc content, but inversely with pH, reducing sugars, radical scavenging activity, nickel, lead, iron, calcium, magnesium, and potassium. Most correlations exhibited again moderate to strong magnitude. Percentage bactericidal activity against clinical *Staphylococcus aureus* isolate revealed primarily moderate correlations, with positive associations in the case of impurity content, total acidity, reducing sugars, iron, manganese, and magnesium, while showing negative associations with nickel. However, we observed fewer significant relationships in the case of the methicillin-resistant clinical strain of this bacterium; that is, weak negative correlations with impurity content, polyphenol content, iron, manganese, and magnesium.

Bactericidal activity against the standard (ATCC) strain of *Streptococcus pneumoniae* revealed positive correlations with ash, copper, chromium, and zinc content, but negative correlations with impurity content, pH, reducing sugars, lead, iron, manganese, magnesium, and potassium. The majority of these associations were moderate to very strong. The correlations observed for the clinical isolate of this species were mainly moderate, i.e., direct associations with nickel, calcium, and potassium, and reverse associations with impurity content, ash content, reducing sugars, polyphenol content, cadmium, zinc, iron, manganese, and magnesium. In the case of the standard *E. coli* strain, the bactericidal effect of honey disclosed moderate to strong relationships with other parameters. Thus, it correlated directly with nickel, calcium, and potassium. On the other hand, negative associations were detected for impurity content, ash content, total acidity, and cadmium, zinc, and manganese contents. The bactericidal effect against the clinical isolate of *E. coli* displayed fewer significant associations, with positive correlations observed for water content, polyphenol content, cadmium, and nickel, and negative correlations identified for copper and calcium.

## 4. Discussion

The antibacterial activity of honey is not yet fully elucidated, owing to its complex chemical composition and its dependence on geographical and botanical origin. Consequently, further studies are warranted, as each honey sample and type exhibits distinct antibacterial properties. The prevailing evidence suggests that the antimicrobial activity of honey predominantly acts against Gram-positive bacteria—including *Staphylococcus aureus*, *Staphylococcus epidermidis*, *Enterococcus faecalis*, *Bacillus subtilis*, *Bacillus cereus*, and certain antibiotic-resistant strains such as methicillin-resistant *S. aureus* (MRSA)—while exerting a comparatively lesser effect on Gram-negative bacteria, such as *Escherichia coli*, *Pseudomonas aeruginosa*, *Enterobacter* spp., *Klebsiella pneumoniae*, *Salmonella enterica*, and *S. typhimurium* [17,19,20,21,49,62,63,64,65,75]. Conversely, a number of studies have reported that Gram-positive bacteria exhibit greater resistance to the antimicrobial action of honey than their Gram-negative counterparts. Furthermore, whilst the majority of the literature supports a demonstrable antibacterial effect of honey, certain studies have documented the absence of such activity for specific honey types against particular bacterial strains [19,49].

The available evidence indicates that the antimicrobial activity of honey is attributable to a combination of physicochemical parameters, including its osmotic effect, low pH, and high sugar concentration, as well as the abundance of compounds with bacteriostatic and bacteriolytic properties, such as lysozyme, hydrogen peroxide, antioxidants, polyphenols, phenolic acids, flavonoids, methylglyoxal, and antimicrobial peptides [56,65,76,77,78].

The comparative analysis of the antibacterial activity of Acacia honey from the 2021 and 2022 harvests revealed significantly greater antibacterial potential in the 2021 production against *Staphylococcus aureus* ATCC 25923, *Escherichia coli* ATCC 25922, and clinical isolates of *Streptococcus pneumoniae*. This enhanced antibacterial activity of the 2021 Acacia honey was consistent across all bacterial strains examined. Statistical analysis of the data obtained in the present study demonstrates that the antimicrobial activity of Acacia honey is dependent on both harvest year and bacterial strain, with particularly pronounced differences observed between *S. aureus* and *S. pneumoniae*. Notably, a more substantial antibiofilm effect against *E. coli* was observed in honey samples from the 2021 harvest.

Also, the analysis of the antibacterial activity of polyfloral honey from the 2021 and 2022 harvests revealed that the 2022 polyfloral honey exhibited significantly greater antibacterial activity against *Staphylococcus aureus* ATCC 25923 and *Streptococcus pneumoniae* ATCC 49619, whilst demonstrating considerably reduced efficacy against methicillin-resistant *Staphylococcus aureus* (MRSA) ATCC 25923. With respect to *Escherichia coli* ATCC 25922, the 2021 polyfloral honey displayed a significantly stronger effect against the standardized strain (ATCC) compared to clinical isolates. Furthermore, polyfloral honey and Acacia honey exhibited distinct antibacterial profiles. Statistical analysis provides evidence that the antimicrobial activity of polyfloral honey is dependent on both harvest year and bacterial strain. Honey samples from the 2021 harvest demonstrated greater efficacy in inhibiting biofilm formation in *Staphylococcus aureus* ATCC 25923 and *Escherichia coli* ATCC 25922, whilst samples from the 2022 harvest exhibited generally stronger bactericidal effects across all bacterial strains included in the study.

The findings of the present study are consistent with those reported in the literature. Several studies have documented variation in the chemical composition—including phenolic compounds, flavonoids, reducing sugars, and related constituents—as well as in the antioxidant, antimicrobial, and anti-inflammatory properties of honey, both across different honey varieties and within the same variety. These variations may be quantitative in nature, but are predominantly qualitative, directly influencing the biological activity of the bioactive compounds present in honey [78,79,80,81]. In the present study, the antibacterial potential of the two honey varieties—polyfloral and Acacia—was assessed on the basis of cell viability testing, with the values obtained compared against those reported in the literature. Antimicrobial activity was classified as weak, moderate, or strong when cell viability values fell within the ranges of 1.0–12.5%, 12.5–50%, and above 50%, respectively, thereby confirming that the honey samples investigated possess measurable antimicrobial properties [82].

The honey varieties tested exhibited distinct antibacterial and antibiofilm profiles. Acacia honey demonstrated significantly greater antibacterial activity against *Staphylococcus aureus* ATCC 25923, *Staphylococcus aureus* ATCC 43300, and both strains of *Streptococcus pneumoniae* ATCC 49619. Polyfloral honey, by contrast, exhibited significantly enhanced biofilm inhibition against methicillin-resistant *Staphylococcus aureus* (MRSA) ATCC 43300, *Staphylococcus aureus* ATCC 25923, and both *E. coli* strains—ATCC 25922 and the clinical isolate. Based on the results obtained and the subsequent statistical analysis, polyfloral honey was found to be superior in preventing biofilm formation for both *Staphylococcus aureus* (Gram-positive) and *E. coli* (Gram-negative) strains. Acacia honey, on the other hand, demonstrated greater bactericidal efficacy, particularly against *Streptococcus pneumoniae* ATCC 49619 and *Staphylococcus aureus* MRSA ATCC 43300.

A study investigating the antimicrobial potential of multiple natural polyfloral honey samples sourced directly from apiaries in Slovakia reported good antibacterial activity against both Gram-positive and Gram-negative bacterial strains, including *S. aureus*, *E. faecalis*, *B. subtilis*, *S. enterica*, and *Y. enterocolitica*, with antibacterial effects found to be both dose- and strain-dependent. The same study also assessed the antibacterial potential of Acacia honey, with the most pronounced effects recorded against *E. faecalis*. The authors concluded that the strongest antimicrobial activity was observed in polyfloral and linden honey samples obtained from beekeepers, and that differences in geographical and botanical origin exerted a significant influence on the antibacterial properties of honey [49].

A further study examined the inhibitory effects of 32 raw honey samples obtained from apiaries—as well as commercially available honey samples of various floral origins, including polyfloral and Acacia honey—on a range of Gram-positive and Gram-negative bacteria. The antibacterial potential of honey was evaluated against eight clinical isolates: *S. aureus*, *E. coli*, *K. pneumoniae*, *P. aeruginosa*, *P. vulgaris, S. typhi*, *Sh. sonnei*, and *S. aureus* MRSA. The findings demonstrated that honey was effective in inhibiting *P. aeruginosa* and *S. aureus* MRSA [83].

The antimicrobial potential of polyfloral and Acacia honey samples sourced from apiaries located in Serbia was further investigated against standardized (ATCC) Gram-positive and Gram-negative bacterial strains, as well as fungal strains. The authors reported minimum inhibitory concentration (MIC) values ranging from 25 mg/mL against B. cereus to 100 mg/mL against *E. coli*, *P. aeruginosa*, *S. typhimurium*, *S. epidermidis*, *K. pneumoniae*, *B. subtilis*, *E. faecalis*, and *Micrococcus lysodeikticus*. The honey samples tested demonstrated both antibacterial and antifungal activity [84].

A related study investigated the antibacterial effect of Spanish honey of varying botanical origins against *S. epidermidis*, demonstrating its capacity to inhibit bacterial growth and induce cell death—an activity attributed to the diversity of botanical origin [56]. Further studies supporting a direct correlation between antimicrobial capacity and the botanical origin of honey have employed bacterial strains including *S. aureus*, *S. epidermidis*, *S. typhimurium*, *E. coli*, and *P. aeruginosa*, with results consistently indicating that the antibacterial activity of honey is dependent on its botanical origin [85,86,87].

Another study examined the antibacterial potential of polyfloral honey samples sourced from apiaries located in Romania, tested against twelve Gram-positive and Gram-negative bacterial strains. Among the strains included in the study, *S. aureus* and *S. epidermidis* were found to be the most susceptible to the antibacterial action of polyfloral honey, followed by *P. fluorescens, B. subtilis*, and *P. aeruginosa*. Nevertheless, certain polyfloral honey samples were found to exert no antibacterial effect against all bacterial strains tested, with some *Salmonella* spp. strains displaying resistance to the action of particular honey samples. Polyfloral honey samples originating from submontane and montane regions proved to be the most effective in terms of antibacterial activity, with geographical and botanical origin directly influencing the antibacterial potential of honey [17]. Evidence from examining the inhibitory and antimicrobial capacity of honey from the Miramar region of Argentina reported high inhibitory and antimicrobial efficacy against *E. coli*, attributing this effect to the influence of the botanical origin of the honey samples analyzed [88].

The antibacterial potential of Acacia honey from the Hail region of Saudi Arabia was assessed against Gram-positive and Gram-negative bacterial strains. The results indicated an inhibitory effect on the growth of nearly all bacterial strains included in the study, with the two clinical isolates—*S. aureus* and *E. coli*—alongside the standardized strain *S. epidermidis* ATCC 12228 proving to be the most susceptible to the action of honey [77]. A study investigating the antibacterial effect of Acacia honey collected in Malaysia evaluated its activity against a broad panel of bacterial strains, including *S. aureus*, *E. coli*, *S. typhimurium*, *P. aeruginosa*, *Listeria monocytogenes*, *Clostridium jejuni*, and *B. cereus*. The authors concluded that Acacia honey exhibits effective antimicrobial activity against the majority of the bacterial strains tested, with particular efficacy against Gram-positive bacteria. Furthermore, the observed variations in the antimicrobial effect of honey were attributed primarily to differences in its chemical composition and phytochemical constituents, which are in turn influenced by botanical origin and physicochemical properties [89].

Based on the results obtained in the present study, a comparative analysis of the two honey varieties revealed that polyfloral honey from the 2021 and 2022 harvests demonstrated the greatest capacity to inhibit bacterial biofilm formation, compared to Acacia honey from the 2022 harvest. Conversely, Acacia honey from both the 2021 and 2022 harvests exhibited a more pronounced antibacterial potential, particularly in comparison to polyfloral honey from the 2021 harvest, which recorded the lowest values for antibacterial potential. Taken together, these findings indicate that polyfloral honey from the 2022 harvest demonstrated the most evident antibacterial activity among all honey samples tested.

A comparative analysis of the antibacterial activity of Acacia and polyfloral honey revealed a more pronounced dose-dependent antimicrobial effect for Acacia honey, whereby increasing honey concentration corresponded to greater antimicrobial efficacy relative to polyfloral honey. In the case of methicillin-resistant bacteria, biofilm inhibition capacity was directly influenced by honey concentration, with a strong relationship observed between the concentration of honey tested and the antibiofilm response obtained. Biofilm inhibition is a physicochemical process—preventing bacterial adhesion to surfaces—and as such, this antibiofilm activity responds more linearly to decreasing honey concentrations than the biologically complex process of cell killing associated with bactericidal effects. Although honey generally exhibited more pronounced antibacterial activity, this effect was associated with greater variability, potentially attributable to bacterial survival mechanisms such as efflux systems or entry into dormant states. Based on the results obtained upon application of the honey samples against methicillin-resistant pathogens, honey appears to have disrupted the highly heterogeneous resistance mechanisms characteristic of this pathogen group, thereby enhancing its antimicrobial effect. From a practical standpoint, this predictable dose–response relationship supports the potential use of honey as an adjuvant or alternative antimicrobial agent against infections caused by methicillin-resistant bacteria, in contexts where conventional antibiotic administration frequently demonstrates variable efficacy.

The findings of the present study are consistent with those reported by other research groups, who have demonstrated that the antimicrobial properties of honey increase with increasing concentration, that the antimicrobial potential of honey is dose-dependent, and that the greatest activity is observed when pure honey is used [49,77,90,91]. However, contrasting evidence exists in the literature, with certain authors concluding that antimicrobial activity was in fact greater when diluted honey (33% *w*/*v*) was used in place of undiluted honey [92]. Analysis of the physicochemical parameters of honey revealed distinct values for each parameter depending on the honey variety and harvest year. Polyfloral honey exhibited the highest impurity content, which may be attributed to the diverse range of pollen present in the honey samples, as well as to the incorporation of particulate matter during collection and processing. Despite this elevated impurity content, the recorded values remained within the limits stipulated by European legislation—which requires that the impurity content of honey not exceed 100 mg/100 g—and are comparable to those reported by Nan et al. (2025) [21], whilst being marginally higher than those documented in a further similar study [20].

Acacia honey recorded the highest pH values among the samples analyzed, whilst polyfloral honey exhibited comparatively lower pH values. A lower pH is associated with greater chemical stability and may contribute to the antimicrobial properties traditionally attributed to monofloral honeys such as Acacia. The pH values recorded in the present study are comparable to those reported in the literature for other honey varieties harvested from apiaries located in Romania [17,19,20,21].

Polyfloral honey recorded higher concentrations of reducing sugars, a finding consistent with increased enzymatic activity and a greater diversity of carbohydrates, reflecting its multiple floral sources. This elevated sugar content influences both the sensory properties and the biological activity of polyfloral honey. Furthermore, polyfloral honey demonstrated a significantly greater DPPH radical scavenging activity and total polyphenol content, findings which support the notion that greater floral diversity—as characterized by polyfloral honey—promotes increased accumulation of phenolic compounds and enhanced free radical scavenging potential.

The mineral content of the analyzed samples varies according to honey variety. Polyfloral honey was notable for its elevated concentrations of magnesium, lead, and manganese, whilst Acacia honey exhibited higher chromium levels; in both cases, however, the values recorded did not exceed the reference limits established by relevant regulatory standards. Overall, polyfloral honey presented a more complex chemical profile, with significantly higher values for several physicochemical parameters associated with nutritional value and antioxidant potential, whilst Acacia honey displayed lower variability and a generally less complex compositional profile.

The honey varieties analyzed in the present study exhibited variability in their macro- and micro-mineral content. The mineral composition of honey is known to be influenced by a range of factors, including geographical origin—encompassing climatic conditions, soil characteristics, and anthropogenic activities—as well as bee species, honey maturation processes, and colony health status, in addition to botanical origin [93,94,95]. The values recorded are consistent with those reported in studies conducted on honey varieties collected from apiaries located in Romania, and collectively indicate the absence of significant pollution sources in the areas where the apiaries are situated [17,19,20,21].

Polyphenol content and DPPH radical scavenging activity (antioxidant activity) emerged as significant factors influencing both biofilm inhibition capacity and antibacterial activity. This indicates that the antimicrobial action of honey is strongly associated with its redox-active compounds, supporting the primary role of phenolic constituents in mediating its antibacterial effect. The metal content of the analyzed honey samples was present in trace quantities, yet exerted an influence on antimicrobial activity in a strain-dependent manner. Certain metals—namely copper, chromium, zinc, nickel, and cadmium—exhibited significant directional correlations and variable enhancement of antibacterial activity. These findings suggest that the metals present in honey do not act uniformly, but are most likely involved in processes such as the augmentation of oxidative stress, interactions with phenolic compounds, or effects on bacterial membrane integrity and enzymatic systems. Furthermore, iron, manganese, and magnesium were found to correlate predominantly negatively with bactericidal effects, suggesting a potential protective or buffering role for bacteria through metabolic or antioxidant pathways.

The polyphenol content and DPPH radical scavenging activity recorded in the present study are comparable to those reported in studies conducted on honey varieties from Romania [19,20,21]. The findings of the present study further corroborate those reported in previous studies, supporting the existence of a close relationship between the phenolic profile of different honey varieties and other bee products, their antioxidant capacity, and their geographical and botanical origin [96,97,98,99,100]. The DPPH radical scavenging activity values recorded for the honey varieties analyzed in the present study were higher than those reported in studies conducted on honey varieties from Spain, Greece, and Croatia, as well as those documented for certain honey varieties from Romania [19,20,21,101,102,103].

Of note, standardized bacterial strains exhibited stronger and more numerous correlations with the chemical parameters of honey than bacterial strains derived from clinical isolates. Specifically, standardized (ATCC) strains demonstrated moderate to very strong associations with the chemical parameters analyzed, whilst clinical isolates displayed fewer and predominantly moderate correlations, suggesting that clinical strains are less chemically sensitive and may possess adaptive mechanisms that reduce their dependence on the chemical composition of honey. Furthermore, variables related to antioxidant properties—namely polyphenol content, DPPH radical scavenging activity, and certain metals with redox-active capacity—showed a tendency to associate with more pronounced bactericidal effects, indicating that oxidative mechanisms play an essential role in the antibacterial action of honey.

One important limitation of the present study is the absence of artificial sugar controls with equivalent osmotic properties. Therefore, the contribution of sugars and osmotic pressure could not be completely separated from the contribution of non-sugar bioactive compounds. Consequently, the observed correlations should be interpreted as exploratory associations reflecting the complex physicochemical profile of honey rather than direct mechanistic relationships. Another drawback of the present study is the relatively limited number of honey concentrations included in the antimicrobial assays. Although the selected concentrations allowed the identification of concentration-dependent trends, a broader dilution range could provide a more detailed characterization of dose–response relationships. In addition, the study was based on honey samples collected from a single geographical region and a limited number of harvest years. Therefore, caution should be exercised when extrapolating the findings to other botanical origins, environmental conditions, or production systems.

## 5. Conclusions

The polyfloral honey analyzed in this study exhibited a more complex chemical profile and higher values across physicochemical parameters, particularly those associated with nutritional value, antioxidant potential, and antibacterial activity. In contrast, Acacia honey displayed a comparatively simpler chemical composition, with lower levels of the investigated parameters.

The chemical composition, antibacterial potential, and biofilm inhibition capacity of both the Acacia and polyfloral honey samples were directly influenced by botanical origin, geographical area, and climatic conditions, as well as by harvesting, processing, and storage practices.

Antibacterial and antibiofilm activity varied considerably between the two honey varieties tested. Polyfloral honey appeared to be more effective in preventing bacterial biofilm formation, achieving inhibition rates of 46.78% (2021 harvest) and 43.63% (2022 harvest) against the clinical isolate *Staphylococcus aureus*, and 44.00% against the Gram-negative bacterium *Escherichia coli* ATCC (2021 harvest). Acacia honey, by contrast, revealed a pronounced bactericidal effect, with inhibition rates of 90.61% against *Enterococcus faecalis* ATCC (2022 harvest), 88.86% against *Shigella flexneri* ATCC (2022 harvest), and 84.97% against a clinical isolate of *Streptococcus pneumoniae* (2022 harvest). Notably, Acacia honey retained significant bactericidal activity even against methicillin-resistant strains, with an inhibition rate of 81.12% against a clinical isolate of *Staphylococcus aureus* MRSA (2022 harvest).

Based on the results obtained for both honey varieties, it can be concluded that honey—through its capacity to inhibit bacterial biofilm formation, eliminate pathogenic bacteria, accelerate wound healing, and reduce inflammation—represents a valuable natural therapeutic agent with an increasingly important role in modern medicine, particularly in the context of rising antimicrobial resistance.

## Figures and Tables

**Table 1:** Palynological analysis of the analyzed Acacia and polyfloral honey samples.

**Honey Sample**		**Acacia (Faba-Ceae)**	**Rosa-Ceae**	**Brasica-Ceae**	**Tilia-Ceae**	**Lamiaceae**	**Astera-Ceae**	**Others**
	**Species**							
Sample A (2021)		47.23%	9.09%	15.33%	4.2%	7.9%	8.16%	8.09%
Sample B (2022)		42.57%	12.1%	17.46%	8.1%	6.35%	7.3%	6.12%
**Honey Sample**		**Astera-Ceae**	**Lamia-Ceae**	**Brassi-Caceae**	**Faba-Ceae**	**Lilia-Ceae**	**Boraginaceae**	**Tilia-Ceae**
	**Species**							
Sample C (2021)		26.42%	12.21%	11.56%	17.48%	8.35%	7.9%	3.94%
Sample D (2022)		21.66%	9.79%	14.39%	19.22%	6.89%	9.2%	2.55%

**Table 2:** Biofilm inhibition capacity of Acacia honey and polyfloral honey.

**Year**	**Honey Type**	**Honey Content**	**SA-ATCC**	**MRSA-ATCC**	**SA-P**	**MRSA-P**
2021	*Acacia*	60%	−5.74 (−6.79; −4.50)	0.90 (0.83; 1.06)	37.52 (35.90; 37.77)	0.46 (0.41; 0.52)
2021	*Acacia*	80%	−9.57 (−9.95; −8.91)	15.71 (14.49; 16.32)	40.02 (39.65; 41.45)	14.84 (14.37; 15.36)
2021	*Acacia*	100%	−18.47 (−19.61; −16.87)	18.74 (17.00; 19.75)	45.52 (45.22; 46.27)	26.98 (25.87; 27.47)
2021	Polyfloral	60%	8.52 (7.38; 9.88)	3.48 (3.16; 3.74)	42.40 (41.97; 42.67)	−10.67 (−11.53; −10.22)
2021	Polyfloral	80%	16.94 (14.94; 17.43)	17.96 (16.71; 18.60)	44.05 (43.67; 44.98)	19.30 (18.42; 19.96)
2021	Polyfloral	100%	27.20 (25.85; 28.33)	20.29 (20.13; 21.87)	46.78 (45.64; 47.02)	28.07 (28.00; 29.32)
2022	*Acacia*	60%	−12.73 (−13.09; −11.85)	4.28 (4.10; 4.38)	34.28 (33.86; 34.79)	10.29 (9.68; 10.67)
2022	*Acacia*	80%	−22.34 (−22.85; −19.29)	6.73 (5.99; 7.36)	37.89 (36.78; 38.14)	11.59 (10.99; 12.59)
2022	*Acacia*	100%	−25.97 (−27.00; −25.90)	13.24 (12.63; 14.07)	41.17 (40.56; 42.21)	29.79 (29.27; 30.71)
2022	Polyfloral	60%	−20.35 (−23.10; −19.59)	15.26 (14.61; 15.76)	23.31 (23.03; 24.10)	15.02 (14.00; 16.41)
2022	Polyfloral	80%	1.82 (1.43; 2.63)	19.19 (18.72; 20.27)	41.25 (40.66; 42.38)	28.62 (28.12; 28.99)
2022	Polyfloral	100%	12.82 (11.04; 13.53)	24.80 (23.17; 26.00)	45.26 (44.45; 46.77)	31.61 (30.13; 32.92)
**Year**	**Honey Type**	**Honey Content**	**SPN-ATCC**	**SPN-P**	**EC-ATCC**	**EC-P**
2021	*Acacia*	60%	−6.86 (−7.04; −6.67)	−16.22 (−17.25; 14.28)	21.71 (21.29; 22.13)	27.36 (26.60; 28.12)
2021	*Acacia*	80%	−7.37 (−8.20; −6.51)	−8.34 (−9.29; −7.54)	22.79 (19.02; 26.56)	29.17 (28.13; 29.71)
2021	*Acacia*	100%	9.14 (8.45; 9.70)	−26.4 (−27.72; −24.97)	27.66 (24.80; 30.52)	30.12 (29.57; 31.67)
2021	Polyfloral	60%	−2.98 (−3.21; −2.62)	−19.25 (−19.93; −18.27)	32.22 (31.53; 32.91)	25.38 (25.16; 26.52)
2021	Polyfloral	80%	−4.33 (−5.53; −4.10)	−12.77 (−13.00; −12.37)	32.41 (29.58; 35.24)	28.38 (28.05; 28.71)
2021	Polyfloral	100%	−7.77 (−8.02; −7.37)	−11.70 (−12.48; −10.90)	44.00 (42.36; 45.64)	34.59 (33.73; 35.45)
2022	*Acacia*	60%	−4.23 (−5.10; −3.13)	−11.57 (−23.28; −9.86)	14.77 (14.22; 16.32)	20.89 (19.71; 21.07)
2022	*Acacia*	80%	4.50 (4.02; 5.37)	−15.56 (−17.59; −13.53)	24.86 (22.49; 27.23)	22.23 (21.15; 22.81)
2022	*Acacia*	100%	12.35 (11.82; 13.70)	−24.87 (−26.97; −22.27)	25.50 (22.72; 28.28)	28.46 (27.06; 29.86)
2022	Polyfloral	60%	3.24 (2.67; 4.42)	−8.64 (−8.94; −7.96)	29.19 (28.52; 29.86)	29.41 (27.98; 29.84)
2022	Polyfloral	80%	11.80 (10.75; 11.94)	−14.10 (−14.66; −13.54)	32.16 (32.03; 32.29)	32.96 (32.10; 33.32)
2022	Polyfloral	100%	12.88 (11.64; 13.32)	−22.61 (−23.16; −22.06)	32.70 (29.37; 34.53)	39.35 (37.36; 41.34)

SA-ATCC, biofilm inhibition capacity (%) against *Staphylococcus aureus* ATCC; MRSA-ATCC, biofilm inhibition capacity (%) against methicillin-resistant *Staphylococcus aureus* ATCC; SA-P, biofilm inhibition capacity (%) against *Staphylococcus aureus* clinical isolate; MRSA-P, biofilm inhibition capacity (%) against methicillin-resistant *Staphylococcus aureus* clinical isolate; SPN-ATCC, biofilm inhibition capacity (%) against *Streptococcus pneumoniae* ATCC; SPN-P, biofilm inhibition capacity (%) against *Streptococcus pneumoniae* clinical isolate; EC-ATCC, biofilm inhibition capacity (%) against *Escherichia coli* ATCC; EC-P, biofilm inhibition capacity (%) against *Escherichia coli* clinical isolate.

**Table 3:** Bactericidal activity of Acacia honey and polyfloral honey.

**Year**	**Honey Type**	**Honey Content**	**SAK-ATCC**	**MRSAK-ATCC**	**SAK-P**	**MRSAK-P**
2021	*Acacia*	60%	60.77 (59.69; 61.86)	65.81 (64.66; 66.97)	61.38 (60.09; 62.67)	64.33 (63.06; 65.59)
2021	*Acacia*	80%	63.21 (62.13; 64.29)	66.48 (65.36; 67.60)	78.47 (77.29; 79.65)	80.47 (79.66; 81.28)
2021	*Acacia*	100%	67.24 (66.31; 68.17)	74.67 (73.90; 75.78)	79.89 (78.66; 81.12)	80.51 (79.73; 81.29)
2021	Polyfloral	60%	50.44 (49.32; 51.56)	63.13 (62.00; 64.27)	69.48 (68.27; 70.69)	65.12 (63.96; 66.28)
2021	Polyfloral	80%	53.07 (52.09; 54.05)	66.63 (65.47; 67.79)	71.64 (70.42; 72.86)	69.71 (68.36; 71.06)
2021	Polyfloral	100%	59.71 (58.62; 60.80)	70.19 (69.30; 71.18)	73.26 (72.34; 74.18)	73.64 (72.47; 74.81)
2022	*Acacia*	60%	72.15 (70.97; 73.33)	53.82 (52.53; 55.11)	51.05 (49.62; 52.47)	69.68 (68.39; 70.97)
2022	*Acacia*	80%	72.86 (71.63; 74.09)	62.53 (61.39; 63.66)	55.63 (54.37; 56.89)	78.06 (76.84; 79.28)
2022	*Acacia*	100%	78.49 (77.38; 79.59)	68.21 (67.31; 69.11)	56.59 (55.44; 57.75)	81.13 (79.97; 82.29)
2022	Polyfloral	60%	68.46 (67.18; 69.74)	51.92 (50.68; 53.16)	66.80 (65.55; 68.05)	66.80 (65.50; 68.10)
2022	Polyfloral	80%	69.04 (67.92; 70.16)	52.95 (51.73; 54.17)	69.45 (68.07; 70.41)	69.24 (67.81; 70.67)
2022	Polyfloral	100%	69.56 (68.44; 70.68)	59.38 (58.29; 60.47)	79.50 (78.45; 80.90)	79.70 (78.55; 80.85)
**Year**	**Honey Type**	**Honey Content**	**SPNK-ATCC**	**SPNK-P**	**ECK-ATCC**	**ECK-P**
2021	*Acacia*	60%	73.93 (72.52; 75.34)	27.15 (25.82; 28.48)	19.67 (18.30; 21.04)	19.87 (18.94; 20.41)
2021	*Acacia*	80%	79.59 (78.36; 80.83)	73.40 (72.50; 74.30)	32.82 (31.46; 34.18)	32.82 (31.73; 33.91)
2021	*Acacia*	100%	80.00 (79.06; 80.94)	80.01 (79.31; 80.72)	65.52 (64.38; 66.66)	65.29 (64.46; 66.58)
2021	Polyfloral	60%	31.06 (29.89; 32.23)	15.06 (13.69; 16.43)	21.55 (20.27; 22.83)	76.78 (75.49; 78.07)
2021	Polyfloral	80%	49.24 (48.12; 50.36)	43.02 (41.73; 44.31)	33.36 (31.88; 34.84)	77.51 (76.22; 78.81)
2021	Polyfloral	100%	58.44 (57.26; 59.62)	49.11 (48.16; 50.06)	44.00 (42.75; 45.25)	77.97 (76.78; 79.16)
2022	*Acacia*	60%	70.12 (68.88; 71.36)	82.34 (80.77; 83.91)	81.35 (80.07; 82.62)	72.72 (71.41; 74.03)
2022	*Acacia*	80%	71.68 (70.44; 72.92)	83.14 (81.76; 84.52)	82.75 (81.42; 84.08)	73.76 (72.54; 74.98)
2022	*Acacia*	100%	72.58 (71.42; 73.74)	84.98 (83.54; 86.42)	88.71 (87.47; 89.95)	76.95 (75.68; 78.22)
2022	Polyfloral	60%	53.42 (52.23; 54.61)	76.20 (74.65; 77.75)	74.36 (73.09; 75.63)	62.73 (61.48; 63.95)
2022	Polyfloral	80%	54.07 (52.84; 55.30)	76.60 (75.20; 78.00)	77.66 ( 76.38; 78.94)	68.68 (66.84; 68.95)
2022	Polyfloral	100%	54.91 (53.72; 56.10)	79.37 (78.14; 80.61)	84.73 (83.50; 85.97)	70.68 (69.27; 71.96)

SAK-ATCC, bactericidal activity (%) against *Staphylococcus aureus* ATCC; MRSAK-ATCC, bactericidal activity (%) against methicillin-resistant *Staphylococcus aureus* ATCC; SAK-P, bactericidal activity (%) against *Staphylococcus aureus* clinical isolate; MRSAK-P, bactericidal activity (%) against methicillin-resistant *Staphylococcus aureus* clinical isolate; SPNK-ATCC, bactericidal activity (%) against *Streptococcus pneumoniae* ATCC; SPNK-P, bactericidal activity (%) against *Streptococcus pneumoniae* clinical isolate; ECK-ATCC, bactericidal activity (%) against *Escherichia coli* ATCC; ECK-P, bactericidal activity (%) of *Escherichia coli* clinical isolate.

**Table 4:** Spearman’s correlation between honey concentration and its antibacterial activity (pooled data 2021–2022).

Bacteriological Parameter	Spearman’s r (*Acacia* Honey)	*p*-Value (*Acacia* Honey)	Spearman’s r (Polyfloral Honey)	*p*-Value (Polyfloral Honey)
SA-ATCC	**−0.73**	<0.001 ***	**0.69**	0.002 **
MRSA-ATCC	**0.81**	<0.001 ***	**0.90**	<0.001 ***
SA-P	**0.86**	<0.001 ***	**0.81**	<0.001 ***
MRSA-P	**0.93**	<0.001 ***	**0.86**	<0.001 ***
SPN-ATCC	**0.76**	<0.001 ***	−0.02	0.917
SPN-P	−0.45	0.065	−0.28	0.245
EC-ATCC	0.57	0.022 *	0.49	0.035
EC-P	0.56	0.023 *	**0.83**	<0.001 ***
SAK-ATCC	0.41	0.087	0.30	0.223
MRSAK-ATCC	**0.73**	<0.001 ***	0.43	0.072
SAK-P	0.40	0.095	**0.70**	0.001 **
MRSAK-P	**0.81**	<0.001 ***	**0.83**	<0.001 ***
SPNK-ATCC	0.40	0.097	**0.66**	0.002 **
SPNK-P	0.38	0.118	0.38	0.119
ECK-ATCC	0.43	0.07	0.45	0.055
ECK-P	0.39	0.10	0.27	0.268

Asterisks (*) indicate significant correlations (Spearman’s correlation, *** *p* < 0.001, ** *p* < 0.01, * *p* < 0.05). Values shown in bold represent coefficients above 0.6. SA-ATCC, biofilm inhibition capacity (%) against *Staphylococcus aureus* ATCC; MRSA-ATCC, biofilm inhibition capacity (%) against methicillin-resistant *Staphylococcus aureus* ATCC; SA-P, biofilm inhibition capacity (%) against *Staphylococcus aureus* clinical isolate; MRSA-P, biofilm inhibition capacity (%) against methicillin-resistant *Staphylococcus aureus* clinical isolate; SPN-ATCC, biofilm inhibition capacity (%) against *Streptococcus pneumoniae* ATCC; SPN-P, biofilm inhibition capacity (%) against *Streptococcus pneumoniae* clinical isolate; EC-ATCC, biofilm inhibition capacity (%) against *Escherichia coli* ATCC; EC-P, biofilm inhibition capacity (%) against *Escherichia coli* clinical isolate; SAK-ATCC, bactericidal activity (%) against *Staphylococcus aureus* ATCC; MRSAK-ATCC, bactericidal activity (%) against methicillin-resistant *Staphylococcus aureus* ATCC; SAK-P, bactericidal activity (%) against *Staphylococcus aureus* clinical isolate; MRSAK-P, bactericidal activity (%) against methicillin-resistant *Staphylococcus aureus* clinical isolate; SPNK-ATCC, bactericidal activity (%) against *Streptococcus pneumoniae* ATCC; SPNK-P, bactericidal activity (%) against *Streptococcus pneumoniae* clinical isolate; ECK-ATCC, bactericidal activity (%)against *Escherichia coli* ATCC; ECK-P, bactericidal activity (%) against *Escherichia coli* clinical isolate.

**Table 5:** Linear regressions for the antibacterial activity of Acacia and polyfloral honey (pooled data 2021–2022).

Honey Type	Bacteriological Parameter	Regression Equation	R^2^
*Acacia*	MRSA-P	y = 57.53x − 30.36	0.869
*Acacia*	MRSA-ATCC	y = 33.06x − 16.65	0.717
*Acacia*	MRSAK-P	y = 34.54x + 48.07	0.717
*Acacia*	SA-P	y = 20.40x + 23.05	0.704
*Acacia*	SPN-ATCC	y = 41.11x − 31.48	0.7
Polyfloral	MRSAK-P	y = 26.78x + 49.28	0.697
Polyfloral	EC-P	y = 24.00x + 12.41	0.669
Polyfloral	MRSA-ATCC	y = 33.93x − 10.23	0.654
Polyfloral	MRSA-P	y = 70.03x − 37.29	0.619
Polyfloral	SAK-P	y = 20.85x + 55.01	0.565
*Acacia*	SA-ATCC	y = −33.47x + 11.31	0.555
*Acacia*	MRSAK-ATCC	y = 29.34x + 41.82	0.528
Polyfloral	SA-ATCC	y = 65.20x − 44.80	0.492
Polyfloral	SA-P	y = 32.45x + 14.68	0.452
Polyfloral	SPNK-ATCC	y = 36.09x + 21.32	0.413
*Acacia*	SA-P	y = 20.40x + 23.05	0.704
*Acacia*	SPN-ATCC	y = 41.11x − 31.48	0.7
Polyfloral	MRSAK-P	y = 26.78x + 49.28	0.697
Polyfloral	EC-P	y = 24.00x + 12.41	0.669
Polyfloral	MRSA-ATCC	y = 33.93x − 10.23	0.654
Polyfloral	MRSA-P	y = 70.03x − 37.29	0.619
Polyfloral	SAK-P	y = 20.85x + 55.01	0.565
*Acacia*	SA-ATCC	y = −33.47x + 11.31	0.555
*Acacia*	MRSAK-ATCC	y = 29.34x + 41.82	0.528
Polyfloral	SA-ATCC	y = 65.20x − 44.80	0.492
Polyfloral	SA-P	y = 32.45x + 14.68	0.452
Polyfloral	SPNK-ATCC	y = 36.09x + 21.32	0.413

R^2^, coefficient of determination; MRSA-P, biofilm inhibition capacity (%) against *methicillin-resistant Staphylococcus aureus* clinical isolate; MRSA-ATCC, biofilm inhibition capacity (%) against *methicillin-resistant Staphylococcus aureus* ATCC strain; MRSAK-P, bactericidal activity (%) against *methicillin-resistant Staphylococcus aureus* clinical isolate; SA-P, biofilm inhibition capacity (%) against *Staphylococcus aureus* clinical isolate; SPN-ATCC, biofilm inhibition capacity (%) against *Streptococcus pneumoniae* ATCC strain; SA-ATCC, biofilm inhibition capacity (%) against *Staphylococcus aureus* ATCC strain; MRSAK-ATCC, bactericidal activity (%) against *methicillin-resistant Staphylococcus aureus* ATCC strain; EC-P, biofilm inhibition capacity (%) against *Escherichia coli* clinical isolate; SAK-P, bactericidal activity (%) against *Staphylococcus aureus* clinical isolate; SPNK-ATCC, bactericidal activity (%) against *Streptococcus pneumoniae* ATCC strain.

**Table 6:** Physicochemical properties of different honey types.

Parameter	*Acacia* Honey (2021)	Polyfloral Honey (2021)	*Acacia* Honey (2022)	Polyfloral Honey (2022)
WC	16.70 (16.59; 16.86)	18.37 (18.34; 18.39)	18.12 (18.11; 18.12)	16.37 (16.37; 16.38)
IC	0.52 (0.52; 0.54)	0.67 (0.67; 0.68)	0.42 (0.41; 0.42)	0.58 (0.56; 0.58)
ASC	0.50 (0.47; 0.53)	0.49 (0.48; 0.50)	0.49 (0.46; 0.50)	0.48 (0.46; 0.48)
HFA	3.77 (3.66; 3.79)	9.11 (9.08; 9.12)	1.50 (1.45; 1.55)	2.60 (2.58; 2.75)
pH	3.01 (3.00; 3.04)	3.27 (3.24; 3.29)	3.15 (3.12; 3.17)	3.35 (3.32; 3.37)
RS	69.43 (68.12; 70.54)	72.05 (71.03; 72.55)	69.83 (68.88; 69.93)	72.68 (71.66; 72.69)
DPPH	47.26 (43.76; 47.42)	49.31 (49.23; 49.31)	44.03 (43.03; 48.64)	53.25 (51.85; 55.35)
PC	808.98 (807.3; 809.2)	1285.7 (1285.5; 1286.1)	960.43 (955.4; 960.7)	1173.11 (1172.5; 1177.6)
Cd	0.04 (0.04; 0.05)	0.06 (0.06; 0.07)	0.04 (0.03; 0.04)	0.03 (0.03; 0.03)
Cu	0.40 (0.39; 0.40)	0.24 (0.24; 0.25)	0.28 (0.27; 0.28)	0.34 (0.34; 0.35)
Ni	0.24 (0.23; 0.24)	0.24 (0.24; 0.25)	0.76 (0.75; 0.77)	0.41 (0.41; 0.42)
Cr	0.11 (0.11; 0.12)	0.09 (0.09; 0.09)	0.09 (0.08; 0.09)	0.07 (0.06; 0.07)
Pb	0.05 (0.05; 0.06)	0.12 (0.11; 0.12)	0.07 (0.06; 0.07)	0.10 (0.10; 0.14)
Zn	10.33 (10.33; 10.55)	9.15 (9.14; 9.20)	4.43 (4.43; 4.50)	3.49 (3.49; 3.58)
Fe	7.80 (7.69; 8.04)	10.57 (10.45; 10.78)	1.08 (1.08; 6.99)	16.34 (16.21; 17.69)
Mn	0.49 (0.47; 0.49)	0.72 (0.72; 0.76)	0.27 (0.27; 0.30)	0.58 (0.57; 0.64)
Ca	56.80 (56.78; 57.81)	48.10 (48.07; 51.06)	202.09 (202.1; 207.1)	257.28 (257.3; 265.8)
Mg	27.97 (25.96; 30.98)	37.10 (34.56; 42.10)	27.36 (22.86; 30.36)	41.67 (39.67; 49.17)
K	119.80 (114.8; 131.3)	162.29 (147.3; 173.8)	873.96 (873.9; 874.0)	946.28 (946.3; 946.3)

WC, water content; IC, impurity content; ASC, ash content; HFA, total acidity of honey; RS, total reducing sugar content; DPPH, antioxidant activity as radical scavenging capacity; PC, polyphenol content; Cd, cadmium content; Cu, copper content; Ni, nickel content; Cr, chromium content; Pb, lead content; Zn, zinc content; Fe, iron content; Mn, manganese content; Ca, calcium content; Mg, magnesium content; K, potassium content.

**Table 7:** Physicochemical properties of different honey types (pooled data 2021–2022).

Parameter	*Acacia* Honey	Polyfloral Honey	*p*-Value
WC	16.86 (16.53–18.00)	17.37 (16.37–18.39)	0.810
IC	0.47 (0.42–0.52)	0.62 (0.55–0.67)	0.010 *
ASC	0.47 (0.45–0.50)	0.48 (0.45–0.50)	0.689
HFA	2.55 (1.53–3.73)	5.98 (2.65–9.09)	0.174
pH	3.09 (3.03–3.14)	3.30 (3.23–3.37)	0.004 **
RS	69.63 (68.21–71.10)	72.37 (71.14–72.69)	0.025 *
DPPH	45.65 (42.03–47.49)	49.88 (49.16–53.25)	0.010 *
PC	884.70 (806.50–958.00)	1233.66 (1172.25–1285.64)	0.004 **
Cd	0.044 (0.036–0.049)	0.046 (0.028–0.062)	0.936
Cu	0.338 (0.281–0.394)	0.247 0.242–0.340)	0.174
Ni	0.495 (0.238–0.756)	0.328 (0.235–0.429)	0.522
Cr	0.106 (0.091–0.118)	0.080 (0.067–0.089)	0.008 **
Pb	0.055 (0.050–0.078)	0.115 (0.103–0.120)	0.006 **
Zn	7.45 (4.45–10.33)	6.41 (3.54–9.15)	0.575
Fe	7.69 (1.08–11.73)	13.51 (10.40–16.28)	0.128
Mn	0.40 (0.27–0.49)	0.66 (0.57–0.73)	0.008 **
Ca	58.30 (56.78–170.77)	155.66 (49.58–257.27)	0.298
Mg	30.68 (24.80–33.84)	39.84 (37.10–45.74)	0.010 *
K	131.31 (112.32–715.94)	565.79 (168.04–946.28)	0.174

Values are given as median with lower and upper quartiles in parentheses. Asterisks (*) indicate significant differences between honey samples obtained from different plant sources (Mann–Whitney U test with Benjamini–Hochberg false discovery rate correction, ** *p* < 0.01, * *p* < 0.05). WC, water content; IC, impurity content; ASC, ash content; HFA, total acidity of honey; RS, total reducing sugar content; DPPH, antioxidant activity as radical scavenging capacity; PC, polyphenol content; Cd, cadmium content; Cu, copper content; Ni, nickel content; Cr, chromium content; Pb, lead content; Zn, zinc content; Fe, iron content; Mn, manganese content; Ca, calcium content; Mg, magnesium content; K, potassium content.

## Data Availability

The data presented in this study are available upon request from the corresponding author.

## Abbreviations

The following abbreviations are used in this manuscript:

ATCC	American Type Culture Collection
CFU	Colony Forming Units
MRSA	Methicillin-Resistant *Staphylococcus aureus*
OD	Optical Density
TSB	Trypticase Soy Broth
DPPH	Antioxidant Capacity
TTC	2,3,5-triphenyltetrazolium chloride
